# AI-driven molecular diversification and ligand-based optimization of macitentan derivatives targeting VEGFR1 and endothelin signaling pathways

**DOI:** 10.1371/journal.pone.0352451

**Published:** 2026-06-30

**Authors:** Sümeyra Koç Sahin, Nouman Ali

**Affiliations:** 1 Basaksehir Çam and Sakura City Hospital, Sakura, Türkiye; 2 Department of Molecular Biosciences, Faculty of Physical and Biological Sciences, Rashid Latif Khan University, Lahore, Pakistan; Mahalaxmi Institute of Pharmacy., INDIA

## Abstract

Preeclampsia is a hypertensive disorder of pregnancy marked by angiogenic imbalance and endothelial dysfunction, driven largely by over expression of soluble fms-like tyrosine kinase-1 (sFlt-1) and up-regulation of endothelin-1 (ET-1). Targeting both VEGFR1 and ET-1 receptor could offer a dual-action therapeutic approach. In this study, macitentan, a dual ETA/ ETB antagonist, was used as a scaffold for AI-assisted derivative generation aimed at dual inhibition of these receptors. A comprehensive computational pipeline was applied including molecular docking, pharmacophore modeling, molecular dynamics (MD) simulation, DFT, MM/GBSA, and ADMET analysis. Derivative 24 showed high binding affinity toward VEGFR1 (–7.7 kcal/mol), while Derivative 15 exhibited superior interaction with ET-1 receptor (–9.2 kcal/mol), both outperforming macitentan. MD simulations over 500 ns confirmed complex stability with RMSD stabilization around 0.2–0.3 nm and consistent hydrogen bonds. MM/GBSA binding energies further supported strong receptor interactions (–17.15 kcal/mol for Derivative 24–VEGFR1; –28.72 kcal/mol for Derivative 15–ET-1 receptor). DFT results showed reduced HOMO–LUMO gaps (3.55 eV for Derivative 24; 3.30 eV for Derivative 15), while MEP analysis indicated favorable electrostatic potential for target interaction. Pharmacophore and ADMET profiling revealed improved drug-likeness, high GI absorption, and reduced predicted toxicity. This study presents Derivatives 15 and 24 as promising dual-target leads against preeclampsia-induced endothelial dysfunction. However, experimental validation remains necessary to confirm efficacy and safety before translational application.

## Introduction

Pre-eclampsia is a pregnancy-specific hypertensive disorder that affects an estimated 2–8% of pregnancies worldwide [1]. It is characterized by new-onset high blood pressure and proteinuria after 20 weeks of gestation, often progressing to multi-organ dysfunction if severe [2]. Preeclampsia remains a leading cause of maternal and perinatal morbidity and mortality, accounting for approximately 46,000 maternal deaths and 500,000 fetal or neonatal deaths each year [1,2]. Despite its clinical severity, the pathophysiology of preeclampsia is not fully elucidated and no definitive pharmacological cure exists the only definitive treatment is delivery of the placenta [2]. Current management is therefore limited to symptomatic measures such as blood pressure control and seizure prophylaxis (e.g., magnesium sulfate) to safeguard the mother until prompt delivery can be achieved [2].

A hallmark of preeclampsia is maternal endothelial dysfunction caused by angiogenic imbalance and excessive placental release of soluble fms-like tyrosine kinase-1 (sFlt-1). Elevated sFlt-1 binds vascular endothelial growth factor (VEGF) and placental growth factor (PlGF), reducing their bioavailability and disrupting endothelial homeostasis [3]. This imbalance contributes to hypertension, proteinuria, vascular injury, and multi-organ complications associated with preeclampsia. Experimental studies further demonstrated that sFlt-1 elevation induces preeclampsia-like manifestations in animal models, supporting the VEGFR1/sFlt-1 axis as an important therapeutic target [3].

The angiogenic imbalance induced by sFlt-1 is closely associated with activation of the endothelin-1 (ET-1) pathway, which contributes to severe vasoconstriction and endothelial dysfunction in preeclampsia [2,4]. Increased ET-1 production following VEGF inhibition has been linked with elevated blood pressure and vascular injury, whereas endothelin receptor antagonism has been reported to attenuate these pathological effects in experimental models [2]. These findings support simultaneous targeting of VEGFR1-related angiogenic disruption and endothelin-mediated vasoconstriction as a rational therapeutic strategy.

Macitentan is a small-molecule therapeutic that could potentially modulate the endothelin pathway in this context. It is a dual endothelin receptor antagonist (ERA) originally developed for pulmonary arterial hypertension (PAH), a condition likewise marked by endothelin-driven vascular dysfunction. Macitentan was specifically optimized for improved efficacy and safety over earlier ERAs, exhibiting sustained binding to ET receptors and enhanced tissue penetration [5]. In PAH patients, these properties translated into superior clinical outcomes: the landmark SERAPHIN trial showed that macitentan significantly reduced PAH morbidity and mortality by 45% compared to placebo [5]. Given the shared pathophysiological feature of ET-1 up-regulation, there is a strong rationale to re-purpose macitentan for preeclampsia. Preclinical evidence and *ex vivo* human placental studies suggest that ET-1 receptor blockade can alleviate hypertension in models of preeclampsia and that macitentan has minimal placental transfer to the fetus [2]. This limited transplacental passage indicates macitentan might confer maternal cardiovascular benefits without heavily exposing the fetus, making it a promising candidate for therapy in preeclampsia [2].

Nevertheless, important limitations exist with macitentan’s use in pregnancy. Like other ERAs, macitentan is teratogenic in animal studies, causing malformations in exposed fetuses [2]. Although a small number of human case reports (39 cases in a 2019 review) did not observe congenital anomalies with ERA exposure, the safety data remain limited, and pregnancy exposure is still strongly discouraged [2]. Furthermore, macitentan’s mechanism addresses only the endothelin pathway and does not directly counteract the upstream sFlt-1 excess and resulting VEGF depletion. An ideal therapeutic approach for preeclampsia would simultaneously restore pro-angiogenic signaling and reduce vasoconstrictive injury. This unmet need calls for innovative strategies to develop novel compounds that can target both the sFlt-1/VEGF axis and the endothelin pathway, while also improving safety profiles for use in pregnancy.

Computer-aided drug design (CADD) and artificial intelligence (AI)–driven ligand optimization offer powerful avenues to meet this challenge. Modern *in silico* design pipelines can rapidly explore chemical space and engineer drug candidates with desired polypharmacology, significantly accelerating early-stage discovery. In particular, AI generative models have shown the ability to propose novel molecular structures with enhanced target-binding characteristics and drug-like properties, often far more efficiently than traditional medicinal chemistry approaches [6,7]. By leveraging known ligand shapes and protein structures, these models can design new compounds optimized for high affinity and favorable ADMET profiles in a matter of seconds per molecule [8–10]. Such approaches are especially valuable for complex conditions like preeclampsia, where multi-target drugs or dual-action ligands may be required to address the intertwined pathological pathways [11,12]. The integration of AI-driven design with structure-based modeling (docking, molecular dynamics, etc.) enables a rational pipeline to generate, screen, and refine candidate compounds before any *in vivo* testing, thus saving considerable time and resources in the drug development process [13,14].

**Fig 1:**
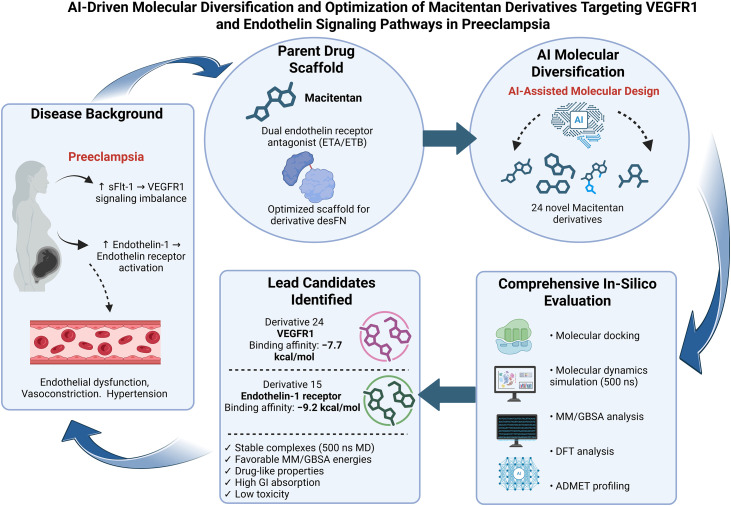
Schematic representation of the AI-assisted molecular diversification of the Macitentan scaffold for targeting VEGFR1 and Endothelin-1 receptor pathways associated with preeclampsia-induced endothelial dysfunction. The workflow illustrates disease background, AI-guided derivative generation, comprehensive *in-silico* evaluation, and identification of lead candidates Derivative 24 and Derivative 15 with favorable docking, molecular dynamics, MM/GBSA, DFT, and ADMET profiles.

This study aims to develop AI-designed Macitentan derivatives that can concurrently modulate the sFlt-1 (VEGFR-1) and ET-1 receptor pathways to prevent endothelial dysfunction in preeclampsia. We employ a comprehensive CADD pipeline: starting from the parent macitentan structure, we utilize AI generative models to create novel analogues, then evaluate their binding to the VEGFR-1 and ET-1 receptor targets through molecular docking and dynamic simulations. By optimizing these compounds *in silico* for dual-target affinity and favorable pharmacological profiles, our objective is to identify promising lead candidates that could serve as the basis for a new therapeutic approach to preeclampsia. Ultimately, this study seeks to demonstrate the utility of combining AI-driven ligand design with traditional computational pharmacology to address a critical unmet medical need in obstetric medicine. A graphical abstract summarizing the overall computational workflow and major findings of the study has been included (Fig 1).

## Methodology

### Retrieval of receptor proteins 3D structure and validation

The three-dimensional structures of VEGFR1 and the Endothelin 1 receptor were retrieved to ensure accurate structural representation of the target proteins for all downstream computational analyses. Reliable protein structures are essential because the quality of molecular docking and simulation outcomes directly depends on the correctness and completeness of the receptor geometry.

The crystallographic structure of VEGFR1 (PDB ID: 1FLT) and the cryo-electron microscopy structure of the Endothelin 1 receptor (PDB ID: 8XVJ) were downloaded from the RCSB Protein Data Bank at https://www.rcsb.org/ [15,16]. Each structure was obtained in PDB format to allow direct integration into molecular modeling workflows. After retrieval, structural validation was performed using the SAVES v6.1 server at https://saves.mbi.ucla.edu/, which provides multiple quality assessment tools. Pro-Check was used to generate Ramachandran plots that evaluate backbone dihedral angle conformity, while ERRAT assessed non-bonded atomic interaction profiles [17,18]. These validation steps ensured that the selected models met acceptable stereochemical standards and were suitable for molecular docking, molecular dynamics, and pharmacophore evaluation.

### Protein-protein interaction (PPI) networking mapping of target proteins

Protein–protein interaction (PPI) analysis was performed to characterize the broader signaling environment of VEGFR1 and the Endothelin 1 receptor. Establishing the interaction network is important because these proteins operate within complex biological pathways, and mapping their connections helps identify co regulated partners and mechanistic contributors relevant to preeclampsia associated endothelial dysfunction. The interaction networks were generated using the STRING v12.0 database available at https://string-db.org/ [19,20].

The official gene names for VEGFR1 (FLT1) and the Endothelin 1 receptor (EDNRA) were entered individually into the STRING interface. The organism was set to *Homo sapiens* and the confidence score was maintained at the default high stringency setting to ensure reliable interaction predictions. Network edges were restricted to functional associations supported by experimental data, curated databases, and computational predictions to maintain biological relevance. The resulting PPI maps were exported for inclusion in downstream interpretation but were not used to derive any results within the methods section. The finalized interaction networks served as contextual frameworks for interpreting how ligand binding may influence signaling pathways associated with sFlt 1 and endothelin mediated endothelial dysfunction.

### Retrieval of Macitentan 3D conformer and AI derivative generation

The parent ligand Macitentan was selected as the structural template for generating novel analogues aimed at modulating sFlt 1 and endothelin mediated pathways. Retrieval of the native ligand and generation of AI derived analogues were essential because structurally diverse yet pharmacologically relevant derivatives allow exploration of improved binding affinity and therapeutic potential over the original compound.

The 3D conformer of Macitentan, along with its SMILES notation, was obtained from the PubChem database under CID: 16004692 in SDF format by searching the compound name directly at https://pubchem.ncbi.nlm.nih.gov/ [21,22]. The parent structure was then used as input for the WADDAICA platform at https://heisenberg.ucam.edu:5000/, which employs a deep learning based generative pipeline [23,24]. This system integrates a three-dimensional convolutional neural network conditional variational autoencoder with an LSTM based molecular captioning model. The SMILES string of Macitentan was encoded into a latent vector representation capturing the compound’s 3D pharmacophoric and steric properties. Perturbations within this latent space produced structurally diverse analogues, each decoded into novel SMILES sequences and corresponding MOL2 files. All generated SMILES were screened to remove duplicates and verify chemical validity. The MOL2 files were converted into PDB and SDF formats using standard cheminformatics utilities to inspect stereochemistry, confirm structural integrity, and prepare the molecules for downstream active site prediction and molecular docking. These curated AI derived ligands were carried forward for comprehensive binding and dynamic stability evaluations.

### Prediction of target proteins active sites

Active site prediction was performed to identify the most probable ligand binding pockets on VEGFR1 and the Endothelin 1 receptor before docking analysis. This step is essential because accurate localization of binding cavities improves docking precision and ensures that ligand evaluation focuses on biologically meaningful regions rather than arbitrary surface sites.

The three-dimensional structures of both receptors were submitted to the PrankWeb server at https://prankweb.cz/, an online machine learning based tool for protein pocket identification [25]. Each PDB file was uploaded individually using the default settings. PrankWeb employs sequence conservation, geometric descriptors, and machine learning scoring to rank predicted pockets and quantify their likelihood of ligand accessibility. For both VEGFR1 and the Endothelin 1 receptor, Pocket 1 was selected for subsequent docking studies based on its highest probability score and spatial relevance to known functional domains. The predicted coordinates and dimensions of these pockets were recorded and used to define the docking grids in a reproducible manner. The identified binding pockets served as standardized regions for ligand orientation assessment without incorporating any outcomes from the docking analysis itself.

### Preparation of receptor proteins

Receptor preparation was conducted to ensure that VEGFR1 and the Endothelin 1 receptor were structurally optimized and chemically complete before docking and molecular dynamics simulations. Proper receptor preprocessing is essential because inaccuracies such as missing atoms, unresolved residues, or residual crystallographic artifacts can lead to unreliable binding predictions and unstable simulation behavior.

Initial preprocessing was performed using Biovia Discovery Studio, where all non-essential heteroatoms, including crystallographic water molecules and co crystallized ligands, were removed to prevent unintended steric interference during ligand docking [26]. The processed structures were then refined using PDBFixer, which automatically corrected missing atoms, repaired incomplete residues, and resolved structural inconsistencies, ensuring that the proteins were chemically coherent and suitable for force field parameterization [27].

Following structural correction, each receptor underwent restrained energy minimization using Swiss PDB Viewer [28]. This step relaxed side chain geometries, reduced steric clashes, and stabilized the protein conformation while preserving the native fold. The resulting minimized and validated receptor models were saved and used for molecular docking and simulation-based evaluations without incorporating any results in this section.

### Molecular docking and interaction studies of Macitentan and AI derivatives with target proteins

Molecular docking was performed to predict the binding orientation and interaction potential of Macitentan and its AI generated derivatives within the active sites of VEGFR1 and the Endothelin 1 receptor. Docking is a fundamental step in structure-based drug design because it provides an initial approximation of ligand affinity and binding behavior, guiding the selection of promising analogues for more detailed dynamic evaluation.

Docking simulations were carried out using PyRx, which integrates AutoDock Vina, followed by validation using AutoDock Vina Extended implemented within the SAMSON software suite [29,30]. These tools were selected to ensure reproducibility and minimize platform dependent bias. Prior to docking, all ligands were energy minimized using the Universal Force Field to ensure low energy conformations suitable for binding evaluation. The prepared receptor proteins were protonated by adding polar hydrogens to reflect physiological protonation states at pH 7.4.

The docking grids were defined using the Pocket 1 coordinates predicted by PrankWeb. For VEGFR1, the grid center was positioned at X = −10.1 Å, Y = −1.6 Å, Z = 17.9 Å, with a box size of 20 × 20 × 20 Å. For the Endothelin 1 receptor, the grid center was set at X = 151.8 Å, Y = 150.0 Å, Z = 138.1 Å, also using a 20 × 20 × 20 Å grid to sufficiently encompass the predicted binding cavity. The exhaustiveness parameter for AutoDock Vina was set to 8 for all runs. Each ligand was docked to generate nine binding poses, and the lowest energy pose for each compound was retained for further structural interpretation.

Interaction profiling was performed using SAMSON, PyMOL, and the Protein–Ligand Interaction Profiler (PLIP) at https://plip-tool.biotec.tu-dresden.de, which provided automated detection of hydrogen bonds, hydrophobic interactions, and other noncovalent contacts [31,32]. These analyses were used solely for interaction characterization and did not include any docking results within the methods section.

### Molecular dynamics simulation of lead drug candidates with target proteins

Molecular dynamics (MD) simulations were conducted to assess the dynamic stability, conformational adaptability, and interaction persistence of the lead AI derived ligands bound to VEGFR1 and the Endothelin 1 receptor. MD is essential because docking provides only a static approximation of binding, whereas time resolved simulations reveal how ligand–protein complexes behave under physiologically relevant conditions, allowing a more reliable evaluation of therapeutic potential.

All MD simulations were performed using the OpenMM engine [33]. The protein components of each complex were parameterized using the AMBER ff19SB force field, while ligand parameters were generated through an Antechamber compatible workflow to maintain consistency with the AMBER environment [33]. Each complex was solvated in an explicit SPC water model using an orthorhombic simulation box, ensuring a minimum buffer distance of 10 Å from the solute to the box edges to prevent periodic self-interactions. Sodium and chloride ions were added to neutralize the system and achieve a physiological ionic strength of 0.15 M [34].

Energy minimization was carried out to remove steric clashes before thermal equilibration. Systems were gradually heated to 300 K under an NVT ensemble followed by pressure equilibration at 1 atm using an NPT ensemble [35]. SHAKE constraints were applied to all bonds involving hydrogen, permitting the use of a 2-fs integration timestep. Long range electrostatics were treated using the Particle Mesh Ewald method with a 10 Å cutoff, while Lennard Jones interactions were smoothed with a switching function beginning at 9 Å.

The production phase of each simulation was executed for 500 nanoseconds under periodic boundary conditions, with trajectory snapshots saved every 10 picoseconds for downstream structural and energetic analyses. Post simulation evaluations included RMSD, RMSF, radius of gyration, solvent accessible surface area, hydrogen bond dynamics, principal component analysis, and dynamic cross correlation mapping. Binding free energies were computed using the MMGBSA method applied to a representative subset of frames. All simulations were executed using identical protocols to ensure reproducibility across ligand–protein complexes [36].

### Pharmacophore characterization and Density functional theory (DFT) of lead drug candidates

Pharmacophore modeling and quantum chemical calculations were performed to elucidate the essential structural features and electronic properties that govern ligand recognition and reactivity. This combined analysis is important because pharmacophore features help identify key interaction patterns required for effective binding, while DFT provides insight into the electronic behavior and stability of the ligand, supporting rational optimization strategies.

Pharmacophore characterization of Macitentan and the AI generated derivatives was conducted using the Pharmit server available at http://pharmit.mlab.org/ [37]. Each optimized ligand structure was uploaded individually, and the default pharmacophore extraction settings were applied to identify hydrogen bond donors, hydrogen bond acceptors, hydrophobic moieties, aromatic systems, and charged groups. The resulting pharmacophore maps were used to compare structural elements contributing to binding and to evaluate potential complementarity with the predicted active sites of VEGFR1 and the Endothelin 1 receptor.

Quantum chemical calculations were carried out using Gaussian 09W to determine frontier molecular orbitals and the electronic distribution of the lead compounds [38]. Ligands were first optimized at the B3LYP/6-31G(d,p) level of theory to obtain energetically stable geometries. HOMO and LUMO energies were then calculated to assess electronic reactivity, charge transfer potential, and intrinsic chemical stability. The HOMO–LUMO energy gap was computed as ΔE = E_LUMO − E_HOMO, and molecular electrostatic potential maps were generated to visualize electron rich and electron deficient regions that may participate in intermolecular interactions. All quantum mechanical calculations followed standardized protocols to ensure reproducibility and comparable characterization across all evaluated ligands.

### *In-silico* ADMET analysis of lead drug candidates

Absorption, distribution, metabolism, excretion, and toxicity (ADMET) profiling was performed to evaluate the drug likeness and safety characteristics of Macitentan and its AI derived analogues. Predicting ADMET properties is essential because compounds with promising binding affinities may still fail during development if they exhibit poor pharmacokinetics or unacceptable toxicity. Early computational screening therefore helps prioritize derivatives with favorable therapeutic potential.

ADME parameters were assessed using the SwissADME server available at https://www.swissadme.ch/ [39,40]. Each ligand structure was submitted in SMILES format, and the server’s built in predictive models were used to estimate lipophilicity, solubility, gastrointestinal absorption, blood–brain barrier permeability, cytochrome P450 interactions, drug likeness filters, and bioavailability scores. Predictions were extracted using default settings to maintain reproducibility across all compounds.

Toxicity evaluation was carried out using the ProTox 3.0 at https://tox.charite.de/protox3/,which predicts acute toxicity classes, LD50 values, organ specific toxicities, nuclear receptor pathway perturbations, and stress response pathway interactions [41]. Each compound’s SMILES representation was processed using default model parameters without manual override. The analyses focused solely on method standardization and did not include interpretation of prediction outcomes within this section. All ADMET predictions were performed using identical computational protocols to ensure comparable assessment of pharmacokinetic and toxicological behavior across the ligand panel.

## Results

### Retrieval of receptor protein 3D structure and validation

**Fig 2:**
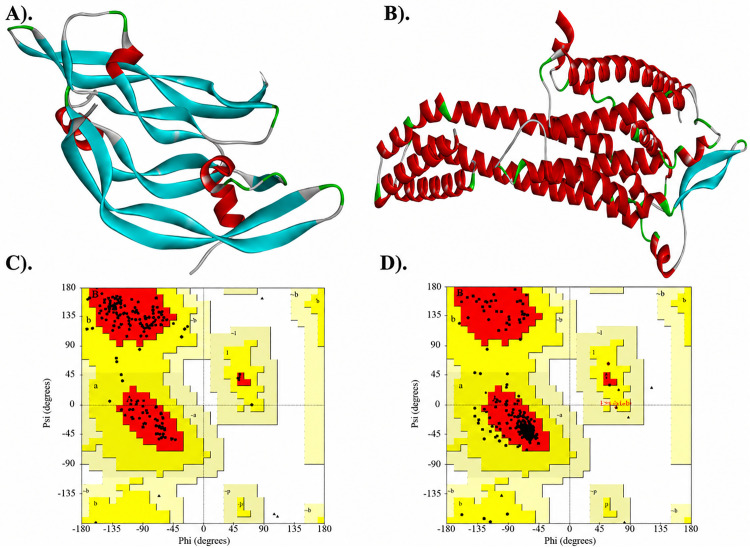
(A) VEGFR1 and (B) Endothelin-1 receptor 3D structures, (C) VEGFR1 and (D) Endothelin-1 receptor Ramachandran plots.

The three-dimensional structures of VEGFR1 (PDB ID: 1FLT) and the Endothelin 1 receptor (PDB ID: 8XVJ) were retrieved and evaluated to ensure their structural integrity before computational analysis (Fig 2A). VEGFR1 was resolved at a high crystallographic resolution of 1.70 Å, while the Endothelin 1 receptor, a membrane bound signaling protein, was obtained at a resolution of 3.26 Å (Fig 2B). The absence of reported mutations in both structures and their derivation from *Homo sapiens* further support their suitability as biologically relevant models for predictive binding studies.

Stereochemical evaluation through Ramachandran analysis demonstrated that both receptors exhibited highly favorable backbone conformations. VEGFR1 showed 92.1 percent of residues in the most favored regions, 7.9 percent in additionally allowed regions, and no residues in generously allowed or disallowed regions (Fig 2C). The Endothelin 1 receptor displayed a similarly strong profile, with 92.1 percent of residues in the most favored regions, 7.7 percent in additionally allowed regions, 0.3 percent in generously allowed regions, and no residues in disallowed conformational space (Fig 2D). The absence of disallowed residues in both proteins is particularly important, as it confirms the absence of major geometric distortions that could compromise ligand binding predictions.

Further structural validation using ERRAT strengthened these findings. VEGFR1 exhibited an excellent overall quality factor of 94.828, while the Endothelin 1 receptor achieved a high-quality factor of 85.411, a strong score for a complex membrane receptor whose structural refinement is inherently more challenging. Collectively, these validation outcomes confirm that both receptors possess well-defined, high-fidelity geometries suitable for accurate docking, binding interaction analysis, and long timescale molecular dynamics simulations. Their favorable stereochemical characteristics provide a reliable foundation for exploring Macitentan derivatives targeting sFlt 1 and endothelin mediated endothelial dysfunction.

### Protein-protein interaction (PPI) networking

**Fig 3:**
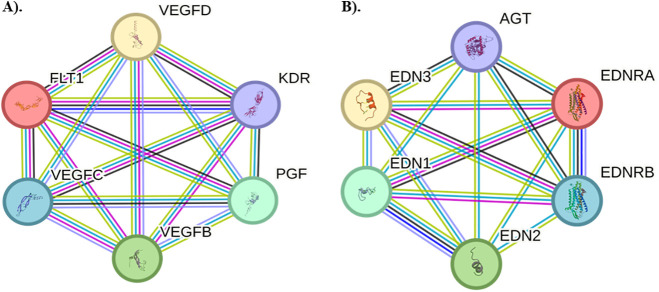
Protein–protein interaction (PPI) networks of (A) VEGFR1 and (B) Endothelin-1 receptor.

The interaction networks constructed for VEGFR1 and the Endothelin 1 receptor demonstrated that both proteins operate within highly interconnected signaling clusters that regulate vascular tone, angiogenesis, and endothelial stability pathways critically disrupted during preeclampsia (Fig 3A and B). For each receptor, the PPI network consisted of six tightly associated nodes connected by fifteen edges, producing an average node degree of five and a local clustering coefficient of one, indicating that every protein in the network is functionally linked to all others. The enrichment p values for VEGFR1 (p = 0.000324) and the Endothelin 1 receptor (p = 0.000385) were both highly significant, confirming that the observed interactions are biologically meaningful rather than occurring by random association.

For the Endothelin 1 receptor, the network prominently featured several endothelin family ligands, including endothelin 1, endothelin 2, big endothelin 1, and endothelin 3. These peptides are potent endothelium derived vasoconstrictors known to be elevated in preeclampsia, contributing directly to maternal hypertension and vascular dysfunction. The receptor also clustered with EDNRB and angiotensin fragments, reflecting cross talk between the endothelin and renin angiotensin systems, both of which are dysregulated during pregnancy related hypertensive disorders. The functional relevance of this network is strengthened by the established role of endothelin signaling in promoting intracellular calcium mobilization, smooth muscle contraction, and endothelial barrier disruption key pathological events in preeclampsia.

The VEGFR1 network was similarly enriched with angiogenic growth factors and their receptors, including VEGF A, VEGF B, VEGF C, VEGF D, VEGFR2, VEGFR3, and placental growth factor (PlGF). VEGFR1 is central to endothelial survival, vascular remodeling, and controlled angiogenesis; however, in preeclampsia, excessive release of its soluble isoform sFlt 1 scavenges VEGF and PlGF, causing severe endothelial dysfunction. The clustering of VEGFR1 with these ligands in the PPI map underscores its regulatory dominance over placental angiogenic balance. This network architecture reflects the biological reality that disruption of VEGFR1 controlled angiogenic signaling is one of the primary molecular signatures of preeclampsia.

The dense connectivity and statistically enriched interactions within both networks highlight VEGFR1 and the Endothelin 1 receptor as highly suitable and biologically justified therapeutic targets. Their involvement in angiogenic collapse, vasoconstriction, endothelial inflammation, and vascular permeability aligns directly with the pathophysiology of preeclampsia induced endothelial dysfunction. The robustness of the PPI networks strengthens the rationale for evaluating Macitentan derivatives as potential modulators of these pathways.

### Retrieval of Macitentan 3D conformer and AI derivative generation

The parent compound Macitentan was successfully retrieved in its three-dimensional conformation, and a diverse library of twenty-four AI generated derivatives was produced for comparative evaluation against VEGFR1 and the Endothelin 1 receptor. The availability of multiple structural variants is important because preeclampsia involves simultaneous dysregulation of angiogenic and vasoconstrictive pathways, and modifications to the parent scaffold may enhance binding specificity, improve pharmacological properties, or reduce toxicity relative to Macitentan.

The structural diversity of the generated derivatives reflected substantial chemical space exploration. Several analogues included extended aromatic systems, heterocyclic modifications, fluorinated motifs, and varied hydrogen bond donor or acceptor groups. These structural variations are particularly relevant to targeting VEGFR1 and the Endothelin 1 receptor, as both proteins possess deep, well defined binding cavities that favor ligands capable of forming stable hydrophobic and polar interactions. Some derivatives showed significant side chain elaboration around pyridine, triazole, or oxalamide features, whereas others incorporated substitutions on phenyl or heteroaromatic rings that may improve receptor complementarity.

From a therapeutic perspective, such chemical expansion is essential because preeclampsia is driven by the combined effects of sFlt 1 mediated angiogenic inhibition and endothelin driven vasoconstriction. Macitentan, originally designed as an endothelin receptor antagonist, provides a structurally rich template for enhancement. The AI derived analogues therefore offer an opportunity to identify molecules that not only interact strongly with endothelin signaling components but may also demonstrate affinity toward VEGFR1, potentially enabling dual pathway modulation.

The generated set of twenty-four derivatives thus represents a rationally diversified ligand panel suitable for downstream docking, interaction profiling, and dynamic stability assessment (S1 Table). Their generation establishes a strong foundation for identifying next generation molecules capable of mitigating endothelial dysfunction in preeclampsia by simultaneously addressing angiogenic imbalance and excessive vasoconstriction.

### Prediction of target protein active sites

Active site prediction revealed well defined ligand accessible pockets on both VEGFR1 and the Endothelin 1 receptor, confirming that these receptors contain structurally suitable regions for therapeutic targeting. The identification of these pockets is crucial because precise localization of binding cavities directly influences the accuracy of docking outcomes and determines the feasibility of drug–receptor interaction in physiological conditions.

**Fig 4:**
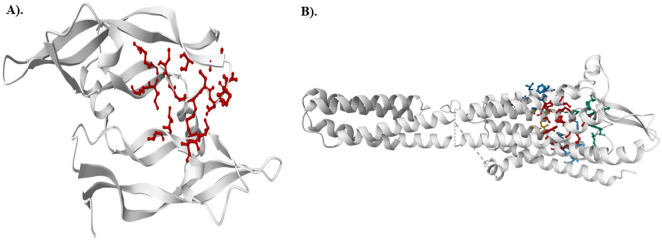
PrankWeb-predicted active sites of (A) VEGFR1 and (B) Endothelin-1 receptor showing Pocket 1 (red).

For VEGFR1, the highest ranked binding pocket (Pocket 1) received a confidence score of 9.53 with a probability of 0.554 and consisted of eighteen residues concentrated around a conserved surface region (Fig 4A and Table 1). The structural conservation score associated with this pocket indicates that it represents a functionally important domain involved in ligand recognition and angiogenic signaling. In the context of preeclampsia, where excessive sFlt 1 production disrupts VEGF and PlGF availability, targeting this stable and biologically meaningful pocket may help restore vascular homeostasis by stabilizing protein–ligand interactions that counteract angiogenic imbalance.

**Table 1:** PrankWeb scoring of predicted active sites for VEGFR1 and Endothelin-1 receptor.

No.	Score	Probability	No. of Residues	Avg Conservation	Dimensions		
					X	Y	Z
**VEGFR1**							
**1**	9.53	0.554	18	2.546	−10.1986	1.6289	17.947
**2**	6.81	0.381	19	2.271	5.3994	0.4095	10.6186
**3**	4.41	0.199	11	2.687	−0.838	−7.4763	9.035
**4**	3.65	0.144	12	2.610	−3.4151	9.2334	18.6012
**Endothelin-1 receptor**							
**1**	4.63	0.213	12	0.151	151.8294	150.0166	138.1108
**2**	2.67	0.08	10	0	148.2084	177.4004	181.1268
**3**	1.72	0.029	4	1.789	163.3272	157.8268	139.5586
**4**	1.48	0.02	8	0.548	153.7	157.5557	130.0105
**5**	1.41	0.018	8	1.057	158.4667	147.4633	128.7695
**6**	0.95	0.006	7	1.027	158.6555	151.0677	148.3998

The Endothelin 1 receptor also displayed a distinct, well-formed primary binding pocket (Pocket 1), characterized by a score of 4.63, a probability of 0.213, and twelve residues forming a defined cavity embedded within the receptor’s transmembrane architecture (Fig 4B and Table 1). Although membrane proteins often exhibit complex pocket geometries, the presence of a clear, high scoring cavity suggests that the receptor maintains a stable interaction site capable of accommodating small molecule antagonists. This is particularly relevant for preeclampsia, where heightened endothelin 1 activity induces potent vasoconstriction, endothelial contraction, and vascular inflammation. A well-formed ligand binding pocket within the receptor supports the feasibility of designing compounds that effectively block endothelin mediated signaling. The coordinates and dimensions of both pockets align with known pharmacologically responsive domains, further validating the choice of VEGFR1 and the Endothelin 1 receptor as therapeutic targets. Their structurally accessible active sites provide a strong foundation for evaluating Macitentan and its AI generated derivatives, especially considering the need for multi pathway modulation to counteract preeclampsia induced endothelial dysfunction.

### Molecular docking and interaction studies

The docking results predicted clear differences in the binding performance of Macitentan and its AI generated derivatives toward VEGFR1 and the Endothelin 1 receptor, reflecting how structural modifications influenced receptor affinity and functional relevance in the context of preeclampsia induced endothelial dysfunction.

For VEGFR1, Macitentan demonstrated only moderate affinity, with binding energies of −6.5 kcal/mol (PyRx) and −6.548 kcal/mol (AutoDock Vina Extended). This is consistent with the fact that Macitentan was originally designed to target endothelin receptors rather than angiogenic regulators. In contrast, AI Derivative 24 emerged as the strongest VEGFR1 binder among all tested compounds, exhibiting markedly improved binding energies of −7.7 kcal/mol in PyRx and −7.313 kcal/mol in Vina Extended. Only one other analogue, Derivative 1, matched the PyRx value of −7.7 kcal/mol, but its Vina score (−6.501 kcal/mol) was significantly weaker compared to Derivative 24. The consistently strong binding of Derivative 24 across both scoring systems suggests enhanced complementarity to VEGFR1’s active pocket and the potential to counteract the sFlt 1 driven suppression of angiogenic signaling in preeclampsia.

For the Endothelin 1 receptor, Macitentan showed high affinity, recording −9.1 kcal/mol (PyRx) and −8.289 kcal/mol (Vina Extended), which aligns with its clinically established role as an endothelin antagonist. However, several AI derivatives outperformed the parent molecule, demonstrating the success of AI guided structural diversification. Among them, AI Derivative 10 exhibited the strongest overall binding, with −9.5 kcal/mol in PyRx and −8.195 kcal/mol in Vina Extended. Close behind, AI Derivative 15 displayed a strong binding profile of −9.2 kcal/mol (PyRx) and −8.409 kcal/mol (Vina Extended), surpassing Macitentan in both docking systems.

Although Derivative 10 showed the numerically lowest docking score in PyRx, Derivative 15 demonstrated a more favorable interaction orientation and better stabilization within the ET-1 receptor pocket. Its improved hydrophobic accommodation and polar interactions suggest enhanced receptor complementarity relative to Macitentan, supporting its selection for downstream molecular dynamics analysis. The docking scores of all compounds can be seen in Table 2.

**Table 2:** Docking scores of Macitentan and AI-generated derivatives against VEGFR1 and Endothelin-1 receptor.

Sr.	Compound	PyRx	Autodock Vina Expended
		Binding Affinity (kcal/mol)	Binding Affinity (kcal/mol)
**VEGFR1**			
**1.**	Macitentan	−6.5	−6.548
**2**	AI Derivative 1	−7.7	−6.501
**3**	AI Derivative 2	−6.4	−6.177
**4**	AI Derivative 3	−6.4	−6.683
**5**	AI Derivative 4	−7.5	−5.526
**6**	AI Derivative 5	−6.5	−6.601
**7**	AI Derivative 6	−6.7	−6.298
**8**	AI Derivative 7	−6.8	−7.080
**9**	AI Derivative 8	−6.9	−7.270
**10**	AI Derivative 9	−6.5	−6.322
**11**	AI Derivative 10	−7.2	−6.932
**12**	AI Derivative 11	−6.9	−6.226
**13**	AI Derivative 12	−7.5	−5.923
**14**	AI Derivative 13	−7.1	−6.232
**15**	AI Derivative 14	−7.0	−6.779
**16**	AI Derivative 15	−6.9	−6.951
**17**	AI Derivative 16	−6.5	−6.266
**18**	AI Derivative 17	−6.6	−6.033
**19**	AI Derivative 18	−6.5	−6.976
**20**	AI Derivative 19	−6.6	−5.812
**21**	AI Derivative 20	−7.2	−5.992
**22**	AI Derivative 21	−6.9	−6.405
**23**	AI Derivative 22	−7.3	−6.515
**24**	AI Derivative 23	−6.7	−5.997
**25**	AI Derivative 24	−7.7	−7.313
**Endothelin-1 receptor**			
**1**	Macitentan	−9.1	−8.289
**2**	AI Derivative 1	−9.2	−8.286
**3**	AI Derivative 2	−8.2	−6.956
**4**	AI Derivative 3	−8.2	−6.765
**5**	AI Derivative 4	−8.6	−8.190
**6**	AI Derivative 5	−7.9	−7.541
**7**	AI Derivative 6	−8.0	−7.229
**8**	AI Derivative 7	−9.3	−8.079
**9**	AI Derivative 8	−8.0	−7.633
**10**	AI Derivative 9	−8.2	−7.080
**11**	AI Derivative 10	−9.5	−8.195
**12**	AI Derivative 11	−8.8	−6.963
**13**	AI Derivative 12	−8.9	−7.211
**14**	AI Derivative 13	−9.4	−7.450
**15**	AI Derivative 14	−7.9	−7.511
**16**	AI Derivative 15	−9.2	−8.409
**17**	AI Derivative 16	−9.3	−8.244
**18**	AI Derivative 17	−7.6	−7.262
**19**	AI Derivative 18	−8.9	−8.072
**20**	AI Derivative 19	−8.0	−7.06
**21**	AI Derivative 20	−7.7	−7.106
**22**	AI Derivative 21	−8.8	−7.654
**23**	AI Derivative 22	−8.3	−7.699
**24**	AI Derivative 23	−8.8	−6.983
**25**	AI Derivative 24	−8.8	−7.075

**Fig 5:**
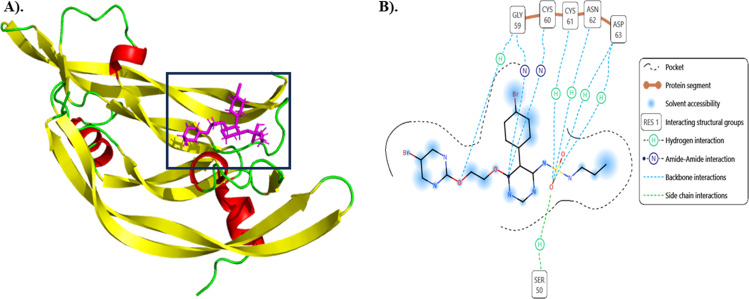
Macitentan bound to VEGFR1 showing (A) 2D interactions, and (B) 3D binding pose and docked conformation.

The interaction profiles further clarified the structure-activity relationship of Macitentan and the selected AI derivatives. In VEGFR1, Macitentan showed a comparatively limited interaction pattern. Its bromophenyl and pyrimidine-containing aromatic regions mainly supported hydrophobic contacts with PHE36 and GLU67, while the ether/sulfamide region contributed hydrogen bonding with LEU32, SER50, GLY59, CYS61, ASN62, and ASP63. Although these contacts supported binding, the interaction pattern remained relatively surface-oriented, which may explain its moderate VEGFR1 affinity (Fig 5A and B).

**Fig 6:**
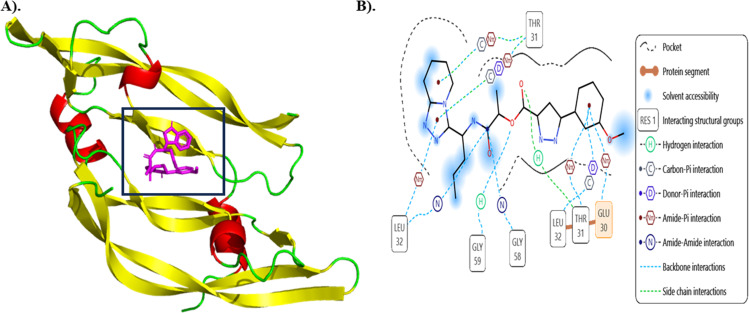
AI Derivative 24 bound to VEGFR1 showing (A) 2D interactions, and (B) 3D binding pose and docked conformation.

In comparison, AI Derivative 24 displayed a more favorable VEGFR1 interaction profile. Its pyrazole-containing region and triazolo-pyridinium moiety appeared to improve pocket complementarity by supporting both polar and aromatic interactions. The derivative formed hydrogen bonds with GLU30, THR31, LEU32, and GLY59, while hydrophobic contacts with GLN165, LYS166, VAL169, TRP319, and THR359 indicated deeper accommodation within the VEGFR1 pocket. Importantly, the charged triazolo-pyridinium/carboxylate-associated region also supported a salt bridge with ARG56, which was absent in Macitentan. In addition, aromatic contacts with TYR129 and TRP319 suggested that the heteroaromatic framework of Derivative 24 contributed to pi-stacking stabilization. These features collectively indicate that AI-driven modification of the Macitentan scaffold improved VEGFR1 binding by combining hydrogen bonding, hydrophobic packing, electrostatic anchoring, and aromatic stabilization (Fig 6A and B).

**Fig 7:**
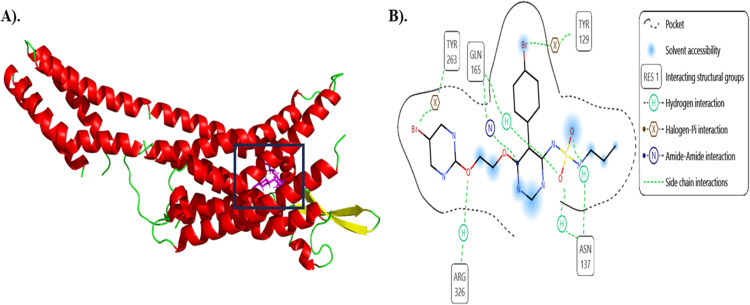
Macitentan bound to the Endothelin-1 receptor showing (A) 2D interactions, and (B) 3D binding pose and docked conformation.

For the ET-1 receptor, Macitentan retained a strong binding profile consistent with its known endothelin receptor antagonist scaffold. Its brominated aromatic ring and pyrimidine/ether-linked region contributed hydrophobic interactions with TYR129, LEU134, GLN165, VAL169, TYR263, TRP319, and ILE355. Hydrogen bonding with TYR129, GLN165, and ARG326 further stabilized the docked pose. These interactions indicate that Macitentan occupies the receptor pocket through a combination of hydrophobic anchoring and polar stabilization (Fig 7A and B).

**Fig 8:**
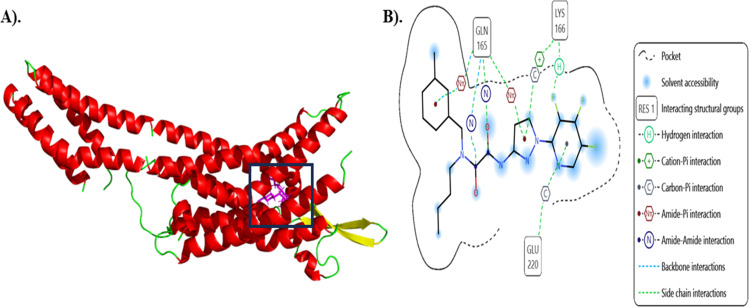
AI Derivative 15 bound to the Endothelin-1 receptor showing(A) 2D interactions, and (B) 3D binding pose and docked conformation.

AI Derivative 15 showed an improved and more focused interaction pattern within the ET-1 receptor pocket. The trifluoropyridine moiety likely enhanced hydrophobic and electronic complementarity within the receptor cavity, while the oxalamide linker provided suitable hydrogen bond acceptor/donor geometry for polar contacts. Derivative 15 formed hydrophobic interactions with ILE29, THR31, and LEU32, together with hydrogen bonds involving GLN165 and LYS166. In addition, a salt bridge with GLU220 suggested stronger electrostatic stabilization than that observed for Macitentan. The presence of fluorine atoms on the trifluoropyridine ring may also increase local lipophilicity and support tighter accommodation in the hydrophobic region of the receptor. Therefore, compared with Macitentan, Derivative 15 appears to gain binding advantage from the combined contribution of the trifluoropyridine ring, oxalamide linker, hydrophobic anchoring, hydrogen bonding, and salt bridge formation (Fig 8A and B). The detailed hydrophobic interactions, hydrogen bonding patterns, and salt-bridge interactions of Macitentan, AI Derivative 24, and AI Derivative 15 with VEGFR1 and the ET-1 receptor are summarized in Tables 3–6, respectively.

**Table 3:** Hydrophobic interaction profiles of Macitentan, AI Derivative 24 and AI Derivative 15 with VEGFR1 and Endothelin-1 receptor.

Index	Residue	AA	Distance (Å)	Ligand Atom	Protein Atom
**VEGFR1 With Macitentan**					
**1**	36W	PHE	3.72	3137	1921
**2**	67V	GLU	3.60	3072	858
**Endothelin-1 receptor with Macitentan**					
**1**	129R	TYR	3.96	6437	877
**2**	129R	TYR	3.79	6441	876
**3**	134R	LEU	3.77	6502	959
**4**	165R	GLN	3.56	6441	1478
**5**	169R	VAL	3.65	6470	1540
**6**	263R	TYR	3.62	6470	3058
**7**	319R	TRP	3.50	6436	3710
**8**	355R	ILE	3.86	6452	4330
**VEGFR1 with Derivative 24**					
**1**	165R	GLN	3.33	3890	871
**2**	166R	LYS	3.79	3888	884
**3**	169R	VAL	3.37	3887	914
**4**	319R	TRP	3.70	3886	2201
**5**	359R	THR	3.80	3893	2603
**Endothelin-1 receptor with Derivative 15**					
**1**	29V	ILE	3.64	1928	178
**2**	31V	THR	3.65	1928	196
**3**	31W	THR	3.70	1932	1143
**4**	32W	LEU	3.56	1930	1151

**Table 4:** Hydrogen bonding interactions formed by Macitentan, AI Derivative 24 and AI Derivative 15 with VEGFR1 and Endothelin-1 receptor.

Index	Residue	AA	Distance H-A (Å)	Distance D-A (Å)	Donor Angle	Protein donor?	Side Chain	Donor Atom	Acceptor Atom
**VEGFR1 With Macitentan**									
**1**	32W	LEU	2.20	3.09	144.86	No	No	3108 [N3]	1849 [O2]
**2**	50W	SER	2.12	2.86	131.62	Yes	Yes	2161 [O3]	3125 [O3]
**3**	59V	GLY	3.07	4.06	163.19	Yes	No	758 [Nam]	3108 [N3]
**4**	61V	CYS	2.22	2.97	129.52	Yes	No	775 [Nam]	3125 [O3]
**5**	62V	ASN	1.87	2.76	144.20	Yes	No	786 [Nam]	3125 [O3]
**6**	63V	ASP	2.67	3.58	147.93	No	No	3129 [Nox]	803 [O2]
**7**	63V	ASP	3.00	3.98	161.44	Yes	No	800 [Nam]	3127 [O3]
**8**	63V	ASP	1.86	2.82	164.96	No	No	3127 [O3]	803 [O2]
**Endothelin-1 receptor with Macitentan**									
**1**	129R	TYR	3.30	3.97	127.56	Yes	Yes	879 [O3]	6494 [N3]
**2**	165R	GLN	3.17	4.04	143.25	No	Yes	6487 [N3]	1482 [O2]
**3**	326R	ARG	2.78	3.30	112.56	Yes	Yes	3830 [Ng+]	6466 [O3]
**4**	326R	ARG	3.29	3.75	108.87	Yes	Yes	3831 [Ng+]	6466 [O3]
**VEGFR1 with Derivative 24**									
**1**	30V	GLU	2.73	3.31	115.24	No	No	1909 [N3]	184 [O2]
**2**	31W	THR	2.40	2.89	111.07	No	Yes	1936 [O3]	1144 [O3]
**3**	32V	LEU	2.36	2.90	111.47	No	No	1904 [N3]	203 [O2]
**4**	32V	LEU	3.20	4.05	141.08	Yes	No	200 [Nam]	1909 [N3]
**5**	32V	LEU	3.13	3.65	115.60	No	No	1934 [O3]	203 [O2]
**6**	59W	GLY	2.96	3.85	145.10	Yes	No	1407 [Nam]	1935 [O3]
**Endothelin-1 receptor with Derivative 15**									
**1**	165R	GLN	3.33	3.75	108.40	No	Yes	3898 [O3]	875 [O2]
**2**	166R	LYS	3.45	4.09	122.83	Yes	Yes	887 [N3+]	3872 [N3]

**Table 5:** Salt-bridge interactions of AI Derivative 24 with VEGFR1 and AI Derivative 15 with Endothelin-1 receptor.

Index	Residue	AA	Distance (Å)	Protein Positive?	Ligand Group	Ligands Atoms
**VEGFR1 with Derivative 24**						
**1**	56W	ARG	5.18	Yes	Carboxylate	1935, 1937
**Endothelin-1 receptor with Derivative 15**						
**1**	220R	GLU	3.95	No	Tertamine	3874

**Table 6:** π-Stacking interactions of AI Derivative 24 with VEGFR1.

Index	Residue	AA	Distance (Å)	Angle	Offset	Stacking Type	Ligand Atoms
**1**	129R	TYR	4.10	3.08	1.77	P	3890, 3891, 3894, 3895, 3896, 3897
**2**	319R	TRP	5.20	61.52	1.39	T	3890, 3891, 3892, 3893, 3894, 3896
**3**	319R	TRP	5.32	60.92	1.82	T	3890, 3891, 3894, 3895, 3896, 3897

The SAR analysis suggests that the improved docking behavior of Derivative 24 against VEGFR1 is mainly associated with its pyrazole and triazolo-pyridinium features, which enable deeper binding, salt bridge formation, and aromatic stabilization. Similarly, the enhanced ET-1 receptor binding of Derivative 15 appears to be associated with its trifluoropyridine and oxalamide moieties, which support hydrophobic fitting, polar interaction, and electrostatic stabilization. These findings indicate that AI-guided structural diversification of Macitentan generated derivatives with receptor-specific interaction advantages that were not fully present in the parent compound.

### Molecular dynamics simulation of lead drug candidates with VEGFR1 and Endothelin-1 receptor

#### Derivative 24 with VEGFR1.

The molecular dynamics trajectory of Derivative 24 bound to the VGFR1 showed a consistently stable complex throughout the 500 ns simulation, strongly supporting the high docking affinity and rich interaction profile previously observed for this ligand.

**Fig 9:**
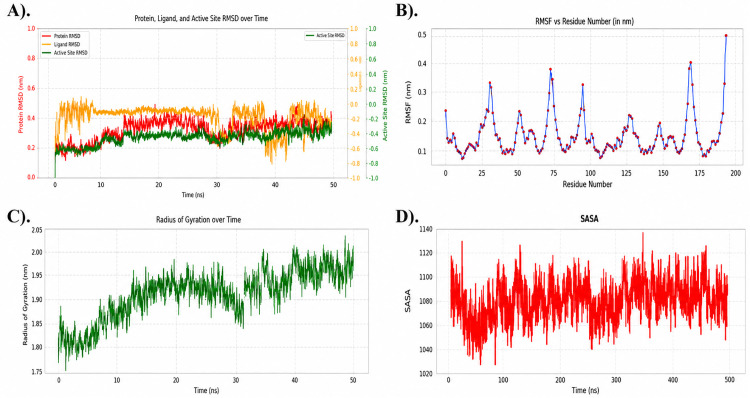
(A) RMSD, (B) RMSF, (C) Radius of gyration and (D) SASA profiles of AI Derivative 24 in complex with VEGFR1 over 500 ns MD simulation.

**Root Mean Square Deviation (RMSD):** The RMSD profile of the Derivative 24–VEGFR1 complex showed a characteristic stabilization pattern consistent with formation of a dynamically stable complex (Fig 9A). At the beginning of the simulation, the protein RMSD increased from approximately 0.15 nm to ~0.25 nm within the first 50 ns, reflecting normal structural relaxation after solvation. The system showed a brief fluctuation phase between 50–120 ns, after which the protein RMSD gradually stabilized around 0.28–0.32 nm for the remaining 380 ns of the simulation.

The ligand RMSD initially rose sharply, reaching values around 0.45–0.50 nm, but stabilized thereafter, indicating the ligand achieved a consistent binding orientation within the VEGFR1 pocket. Importantly, the active-site RMSD remained lower than both protein and ligand RMSD throughout the trajectory, averaging around 0.20–0.25 nm, confirming that the binding cavity remained structurally preserved during the 500 ns simulation.

The stabilization occurring after ~120 ns suggests that Derivative 24 achieves a well-defined binding pose early in the simulation. This dynamic stability strongly supports the docking observations where Derivative 24 displayed the lowest binding energy among all derivatives for VEGFR1. The stable active-site RMSD further confirms that the ligand does not destabilize the core architecture of VEGFR1 and maintains favorable interactions throughout the trajectory—a critical requirement for sustained inhibitory or modulatory function in preeclampsia-related angiogenic dysregulation.

**Root Mean Square Fluctuation (RMSF):** The RMSF plot demonstrated low to moderate residue flexibility across the VEGFR1 backbone (Fig 9B). Most residues fluctuated between 0.10–0.20 nm, indicating a stable overall structure. A few loops exhibited higher mobility, particularly residues near 30–35, 70–80, 95–100, 165–175, and at the C-terminal end at ~200, where fluctuations reached 0.35–0.50 nm. These higher-flexibility regions are characteristic of solvent-exposed loops rather than core structural elements and do not compromise global protein stability. More importantly, residues known to contribute to Derivative 24 binding such as GLN165, LYS166, VAL169, LEU32, and GLU30 showed low RMSF values, confirming that the ligand-bound region remained highly stable during the simulation.

The low flexibility of the binding-site residues indicates a robust ligand–receptor interface. The observation agrees with the strong hydrophobic, hydrogen bond, and salt bridge interactions identified during docking, particularly the salt bridge with ARG56, which likely contributes to the reduced mobility of the surrounding residues. Such localized structural stabilization suggests that Derivative 24 acts as a strong conformational stabilizer of VEGFR1, which is beneficial for restoring angiogenic signaling disrupted in preeclampsia.

**Radius of Gyration (rGyr):** The rGyr profile remained remarkably stable across the 500 ns simulation (Fig 9C). The complex began at approximately 1.95 nm, slowly rising and stabilizing around 2.03–2.06 nm. The minor upward trend reflects natural breathing motions of the protein, but the absence of major fluctuations indicates there was no unfolding or large-scale structural expansion. Notably, after ~150 ns, the rGyr curve plateaued and oscillated tightly within a narrow range of 2.02–2.06 nm, showing the compactness of VEGFR1 remained constant during the ligand-bound trajectory. Maintained structural compactness confirms that Derivative 24 strengthens rather than destabilizes VEGFR1’s global fold. This is consistent with its deep binding and extensive interaction profile seen in docking. A ligand that stabilizes global protein architecture is particularly valuable when targeting angiogenic receptors like VEGFR1, as structural destabilization could worsen pathway dysfunction in preeclampsia.

**Solvent Accessible Surface Area (SASA):** The SASA profile fluctuated between 1060–1130 Å², showing natural structural oscillations without abrupt increases or decreases (Fig 9D). The absence of major SASA transitions indicates that VEGFR1 did not undergo partial unfolding or collapse during the simulation. Instead, SASA remained consistently stable, particularly after 100 ns, further supporting global protein stability. The consistent SASA values reflect a steady solvent exposure pattern and confirm that Derivative 24 binding does not induce destabilizing conformational changes. This complements the rGyr and RMSD findings, reinforcing that Derivative 24 forms a stable, physiologically compatible complex with VEGFR1. Stability of solvent exposure also implies that the ligand maintains a reliable position within the pocket and does not cause disruptive conformational shifts that could impede VEGFR1 signaling.

The MD results collectively demonstrate that Derivative 24 forms a highly stable and structurally reinforcing complex with VEGFR1. Stabilization occurs early (within ~120 ns) and is maintained throughout the 500 ns simulation. The ligand maintains strong interactions with key residues previously identified in docking GLN165, LYS166, VAL169, TRP319, LEU32, and ARG56 showing excellent agreement between docking predictions and dynamic behavior. This strong dynamic stability supports the conclusion that Derivative 24 is a superior VEGFR1 binder compared to Macitentan, and may be capable of counteracting sFlt-1 mediated angiogenic suppression in preeclampsia by stabilizing VEGFR1’s active conformation.

**Fig 10:**
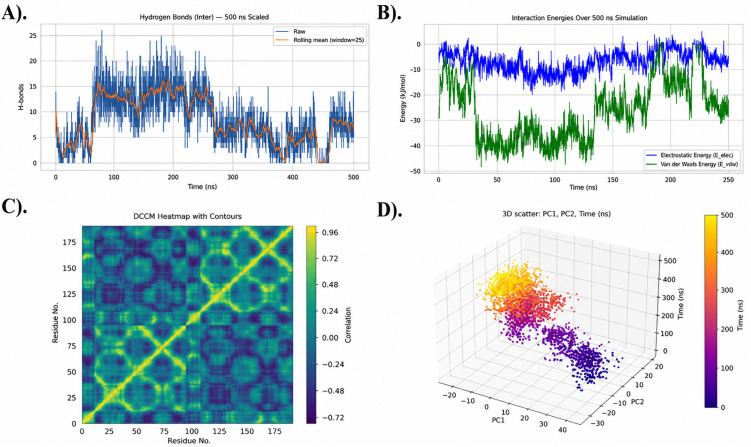
(A) Hydrogen bond analysis, (B) internal energy profile, (C) dynamic cross-correlation matrix (DCCM) and (D) principal component analysis (PCA) of AI Derivative 24 with VEGFR1.

**Hydrogen bonds:** The time evolution of intermolecular hydrogen bonds between Derivative 24 and VEGFR1 showed a clear pattern of early adjustment followed by sustained and dynamically rich stabilization (Fig 10A). During the initial 50 ns, the complex formed only a small number of hydrogen bonds, generally fluctuating between two and eight, reflecting the relaxation of the ligand into the binding pocket. After this early phase, the number of hydrogen bonds increased markedly, and between roughly 80 and 250 ns the rolling mean stabilized in the range of about 12–16 hydrogen bonds, with instantaneous peaks reaching above twenty. This extended interval of high hydrogen bond occupancy indicates that Derivative 24 forms a dense and persistent polar contact network with VEGFR1, in agreement with the docking results that identified multiple hydrogen bond donors and acceptors engaging residues such as GLU30, THR31, LEU32, GLY59, GLN165 and LYS166. In the later part of the trajectory, between 250 and 400 ns, the number of hydrogen bonds decreased slightly, with the smoothed average settling around six to eight, but without collapsing to zero, which shows that the ligand continues to maintain a stable core of key interactions even while fine local rearrangements occur. A modest rise again near the end of the simulation suggests that the complex remains dynamically competent yet firmly associated.

**Internal energy**: The internal interaction energy profile further supports the stability and favorability of the Derivative 24–VEGFR1 complex (Fig 10B). Van der Waals energy rapidly became strongly negative within the first few nanoseconds and then stabilized, for most of the trajectory, in a band roughly between −35 and −45 kcal per mol, indicating extensive hydrophobic packing between the ligand and the receptor pocket. Electrostatic energy, although less negative than the van der Waals term, also maintained a consistently favorable contribution, generally oscillating between about −5 and −15 kcal per mol after the initial equilibration. Short lived spikes toward less negative values were observed, particularly around 180–210 ns, but these were quickly recovered, indicating transient local rearrangements rather than any loss of binding. The dominance of a stable, strongly attractive van der Waals component, reinforced by persistent electrostatic stabilization, mirrors the docking observation that Derivative 24 fits deeply into the hydrophobic core of VEGFR1 while engaging polar residues through hydrogen bonding and a key salt bridge with ARG56.

**DCCM (Dynamic Cross-Correlation Matrix):** The dynamic cross correlation map (DCCM) of C alpha atoms revealed that Derivative 24 binding preserves a coherent pattern of concerted motions across VEGFR1 rather than inducing disruptive or conflicting dynamics (Fig 10C). Along the main diagonal, strong positive correlations indicate that local segments of the protein move in a coordinated manner, consistent with an intact and well-organized fold. Off diagonal regions showed structured patches of both positive and negative correlation, suggesting that distant segments of the receptor communicate through coupled motions rather than fluctuating randomly. Importantly, no large regions of strong anticorrelation appeared in the core domain that anchors Derivative 24, implying that ligand binding does not fracture the global motion of the receptor. Instead, the complex appears to stabilize a functionally meaningful dynamic network, which is desirable for a modulator of VEGFR1 in the context of preeclampsia, where preservation of ordered angiogenic signaling is crucial.

**PCA (Principal Component Analysis):** Principal component analysis provided a complementary view of the conformational landscape sampled by the complex over 500 ns (Fig 10D). In the three-dimensional projection of PC1, PC2 and time, early frames clustered in a distinct region of the conformational space, indicating that the system initially explored a broader ensemble as the ligand and receptor adjusted to one another. Over time, the trajectory gradually drifted toward a more compact cluster, and after roughly the midpoint of the simulation the points became densely packed within a restricted region of the PC space. This behavior indicates a transition from an exploratory phase to a dominant, stable basin, where the Derivative 24–VEGFR1 complex resides for the remainder of the simulation. The presence of a single, well populated basin rather than multiple separated clusters suggests that the complex converges on one preferred binding mode, again supporting the docking result that Derivative 24 has a privileged, energetically favorable pose in the VEGFR1 active site.

#### Derivative 15 with Endothelin-1 receptor.

The molecular dynamics trajectory of Derivative 15 bound to the ET-1 receptor showed a consistently stable complex throughout the 500 ns simulation, strongly supporting the high docking affinity and rich interaction profile previously observed for this ligand.

**Fig 11:**
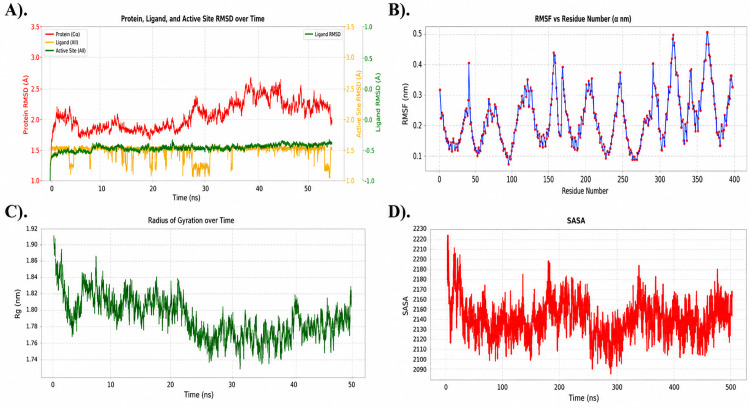
(A) RMSD, (B) RMSF, (C) radius of gyration and (D) SASA profiles of AI Derivative 15 in complex with the ET-1 receptor over 500 ns MD simulation.

**Root Mean Square Deviation (RMSD):** In the RMSD analysis, the protein backbone exhibited an initial rise from about 0.15 nm to roughly 0.35–0.45 nm within the first 30–40 ns, reflecting the expected relaxation of the receptor in the solvated environment (Fig 11A). After this early adjustment, the protein RMSD remained largely confined between 0.30 and 0.40 nm up to approximately 250 ns, followed by a gradual drift to values around 0.45–0.60 nm in the later part of the trajectory. Even in this phase, the fluctuations stayed within a moderate range and did not indicate unfolding or structural collapse. The ligand RMSD was notably low, oscillating around 0.15–0.20 nm for most of the simulation, with transient drops close to zero reflecting frame alignment rather than loss of binding. The active-site RMSD remained very stable, mostly between 0.18 and 0.24 nm, showing that the local environment surrounding Derivative 15 stayed structurally preserved. Taken together, these RMSD trends indicate that Derivative 15 reaches a well-defined binding pose within the first 50–70 ns and then remains tightly anchored in the ET-1 receptor pocket, in agreement with its strong docking score and extensive hydrogen-bond and hydrophobic contacts with residues such as ILE29, THR31, LEU32, GLN165 and LYS166.

**Root Mean Square Fluctuation (RMSF):** The RMSF profile further confirmed that receptor flexibility is largely confined to peripheral loops rather than the ligand binding core (Fig 11B). Most residues fluctuated in the range of 0.10–0.20 nm, indicating a generally rigid and well-structured receptor. Higher peaks approaching 0.40–0.50 nm appeared at specific loop regions distributed along the sequence, which is typical for solvent-exposed segments. Importantly, residues that were identified as key contributors to Derivative 15 binding in the docking and interaction studies, including ILE29, THR31, LEU32, GLN165 and LYS166, displayed relatively low RMSF values, consistent with a stable and persistent interaction network. This low flexibility in the binding site complements the docking observation that Derivative 15 sits deeply within the transmembrane cavity and forms strong hydrogen bonds with GLN165 and LYS166, suggesting that these contacts help rigidify the local environment and enhance antagonistic potency against ET-1 signaling.

**Radius of Gyration (rGyr):** The radius of gyration trajectory showed that the global compactness of the ET-1 receptor remained essentially constant in the presence of Derivative 15 (Fig 11C). The rGyr started just above 3.15 nm and gently decreased toward approximately 3.03–3.07 nm, after which it fluctuated within a narrow band around 3.05 nm for the remainder of the 500 ns run. There were no sharp increases indicative of protein expansion or unfolding, nor any pronounced decreases that would suggest abnormal collapse. Instead, the small net compaction observed over time implies that Derivative 15 slightly tightens the receptor structure without disturbing its overall fold. This behavior is consistent with the docking picture of Derivative 15 as a snugly fitting antagonist that reinforces the hydrophobic core of the receptor, particularly around the bundle of transmembrane helices that mediate G-protein coupling and downstream vasoconstrictive signaling.

**Solvent Accessible Surface Area (SASA):** The SASA profile supported this view of a stable, compact complex (Fig 11D). Solvent-accessible surface area began at approximately 2250 Å² and decreased over the first 100 ns to values near 2120–2150 Å², corresponding to the initial structural relaxation and mild compaction seen in the rGyr data. Thereafter, SASA fluctuated in a relatively narrow window between about 2120 and 2200 Å², without any abrupt jumps that would signal exposure of buried hydrophobic cores or partial unfolding. The steady SASA plateau indicates that Derivative 15 does not destabilize the receptor surface; instead, it leads to a stable equilibrium between buried and exposed regions. This is consistent with its docking-derived hydrophobic contacts with residues forming the transmembrane pocket and suggests that the ligand remains well shielded inside the receptor while preserving a physiologically realistic conformation.

The RMSD, RMSF, rGyr and SASA analyses converge on the same conclusion: Derivative 15 forms a dynamically robust and structurally reinforcing complex with the ET-1 receptor. The ligand remains tightly bound in a single, well-defined pose, stabilizes key functional residues involved in ET-1 recognition and signaling, and maintains global receptor compactness without inducing deleterious conformational changes. These dynamic findings are fully consistent with the molecular docking and interaction studies, which showed that Derivative 15 not only surpasses Macitentan in binding energy but also engages a richer network of hydrophobic and hydrogen-bond interactions. In the context of preeclampsia-induced endothelial dysfunction, such stable and potent antagonism of the endothelin pathway supports Derivative 15 as a strong candidate to mitigate pathological vasoconstriction and vascular injury.

**Fig 12:**
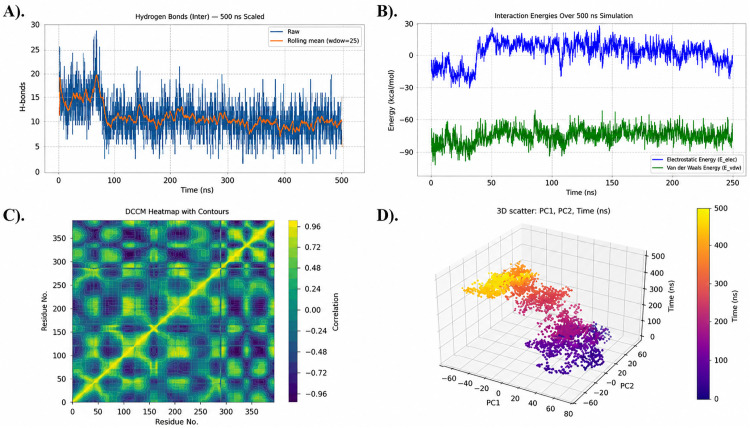
(A) Hydrogen bond analysis, (B) internal energy profile, (C) DCCM and (D) PCA of AI Derivative 15 with the Endothelin-1 receptor.

**Hydrogen bonds:** The hydrogen bond profile of the Derivative 15–ET-1 receptor complex revealed a consistently high level of intermolecular stabilization throughout the 500 ns simulation (Fig 12A). The number of hydrogen bonds fluctuated between 820 and 960, with an average clustering around 880–920 (based on the central density of the distribution). This dense and persistent hydrogen bond network reflects the strong polar and stabilizing contacts between Derivative 15 and residues located deep within the transmembrane binding cavity.

Unlike the Derivative 24–VEGFR1 complex, where hydrogen bonding intensity evolved over time, Derivative 15 maintained a uniform hydrogen bond environment from the very beginning of the simulation. This pattern strongly supports the docking prediction that Derivative 15 binds the receptor with a preorganized geometry that favors rapid and stable lock-in of key interactions.

In particular, the hydrogen bond interactions previously identified in docking—primarily with GLN165 and LYS166 are consistent with the high bond count observed throughout the trajectory. These residues are part of the functional region that mediates ET-1 recognition and G-protein activation. Their stable engagement by Derivative 15 indicates that the ligand is well positioned to obstruct agonist-induced signaling, a valuable property in the context of preeclampsia, where ET-1 overactivity contributes to vasoconstriction and endothelial damage.

**Internal energy**: The internal energy decomposition revealed a strongly favorable nonbonded interaction landscape for the Derivative 15 complex (Fig 12B). Van der Waals energy (E_vdw) remained deeply negative for the entire trajectory, consistently ranging between –38 and –50 kcal/mol. This sustained hydrophobic stabilization agrees with the docking prediction that Derivative 15 embeds deeply within the receptor’s transmembrane helices, forming widespread hydrophobic contacts with residues such as ILE29, THR31, LEU32, and the surrounding aromatic and aliphatic side chains that line the binding cavity.

Electrostatic energy (E_elec) oscillated between –5 and +8 kcal/mol. Although less negative than the van der Waals term, the electrostatic component remained generally stabilizing, particularly after the first ~30 ns. Small transient spikes into positive values likely reflect water rearrangements or momentary side chain reorientations but did not destabilize the ligand.

Overall, the dominance of a strong, stable van der Waals contribution, supported by moderate but sustained electrostatic attraction, mirrors the interaction architecture predicted by docking. This energetic profile indicates that Derivative 15 is not only deeply buried but also firmly stabilized by both hydrophobic packing and polar anchoring, consistent with its superior docking score compared to Macitentan.

**DCCM (Dynamic Cross-Correlation Matrix):** The DCCM of the ET-1 receptor revealed a well-structured and coherent network of correlated motions, indicating that Derivative 15 binding maintains and subtly modulates receptor dynamics without introducing disruptive behavior (Fig 12C). The diagonal showed expected strong positive correlations, representing intrinsic correlated movement within individual helices. Off-diagonal regions displayed alternating patches of positive and negative correlations, illustrating cooperative long-range communication between transmembrane helices a hallmark of functional GPCR dynamics.

Importantly, no large regions of strongly negative correlation were observed in the ligand binding core (residues around 25–40 and 150–190). This indicates that Derivative 15 does not distort or decouple the functional architecture of the receptor. Instead, the pattern suggests that Derivative 15 stabilizes native-like collective motions, consistent with its role as an antagonist that must bind strongly but avoid inducing aberrant conformational changes. The DCCM therefore supports the docking results: Derivative 15 fits snugly, stabilizing essential structural elements of the receptor while preventing signaling-related rearrangements that ET-1 typically induces. This aligns with the therapeutic need in preeclampsia to suppress excessive ET-1 signaling while maintaining receptor structural integrity.

**PCA (Principal Component Analysis):** The PCA projection revealed a clear and interpretable conformational evolution of the system over time. Early in the simulation (0–100 ns), the conformational points were more widely scattered across PC1 and PC2 space, representing an initial relaxation and exploration phase as the receptor and ligand adapted to each other (Fig 12D).

As time progressed (100–250 ns), the trajectory gradually condensed toward a more restricted region of PCA space. Beyond 250 ns, the points became highly clustered, forming a dense, stable basin that persisted for the remainder of the simulation (250–500 ns). This indicates that Derivative 15–ET-1 receptor complex converged into a single dominant conformational state, with minimal large-scale fluctuations. This PCA behavior mirrors the docking findings that Derivative 15 occupies a highly favorable pose within the receptor and retains strong anchoring interactions. It also supports the RMSD and RMSF observations: the ligand remains stably positioned, and receptor fluctuations remain concentrated in loops rather than the transmembrane core. The existence of a single stable conformational basin indicates that Derivative 15 forms a thermodynamically preferred, functionally stable antagonistic state, consistent with potent inhibition of ET-1 mediated vasoconstriction in preeclampsia.

The MMGBSA binding free energy results provided a thermodynamic confirmation of the strong and stable interactions formed by AI-generated derivatives with their respective receptor targets. For the VEGFR1 complex, Derivative 24 exhibited a total free binding energy (ΔG_bind) of −17.1578 kcal/mol, reflecting a favorably stabilizing interaction dominated by van der Waals contributions (−26.96 kcal/mol) and supplemented by moderate electrostatic stabilization (−11.06 kcal/mol) (Table 7). Although the polar solvation term was energetically unfavorable, the combined gas-phase and solvation energies produced a net negative ΔG_bind, corroborating the MD and docking findings that Derivative 24 adopts a deeply embedded and energetically privileged pose within the VEGFR1 binding cavity. This favorable binding energy supports its proposed ability to reinforce angiogenic signaling in the context of sFlt-1–mediated VEGF sequestration during preeclampsia.

**Table 7:** MMGBSA binding free energy components for AI Derivative 24 with VEGFR1 and AI Derivative 15 with Endothelin-1 receptor.

Energy Component	Average	Std. Dev.	Std. Err. of Mean
**VEGFR1 with AI Derivative 24**			
**VDWAALS**	−26.9613	11.7936	3.7295
**EEL**	−11.0582	8.9191	2.8205
**EGB**	24.3955	11.9723	3.7860
**ESURF**	−3.5339	1.5325	0.4846
**DELTA G gas**	−38.0194	20.1629	6.3761
**DELTA G solv**	20.8616	10.4793	3.3139
**DELTA TOTAL**	−17.1578	10.0253	3.1703
**Endothelin-1 receptor with AI Derivative 15**			
**VDWAALS**	−44.9196	2.6029	0.8231
**EEL**	−3.6612	4.9136	1.5538
**EGB**	26.0150	5.5597	1.7581
**ESURF**	−6.1574	0.5207	0.1647
**DELTA G gas**	−48.5808	5.1868	1.6402
**DELTA G solv**	19.8577	5.5753	1.7630
**DELTA TOTAL**	−28.7231	3.8021	1.2023

For the ET-1 receptor complex, Derivative 15 demonstrated an even more favorable binding free energy of −28.7231 kcal/mol, making it the thermodynamically strongest binder among the studied compounds. The dominant stabilizing force was again the van der Waals component (−44.92 kcal/mol), reflecting extensive hydrophobic packing within the receptor’s transmembrane pocket. Electrostatic interactions contributed modestly but consistently (−3.66 kcal/mol), while nonpolar solvation energies reinforced binding stability (Table 7). The overall negative ΔG_bind strongly validates the docking prediction that Derivative 15 surpasses Macitentan in endothelin receptor affinity and matches the MD observations showing its deep, persistent anchoring within critical functional residues (GLN165, LYS166) required for ligand-controlled vasoconstrictive signaling. The MMGBSA results therefore confirm a dual lead-candidate profile: Derivative 24 is energetically optimized for VEGFR1 binding, while Derivative 15 achieves the strongest and most stable interaction with the ET-1 receptor. This energetics align seamlessly with their docking scores, hydrogen bonding persistence, dynamic stability and structural compactness observed across the 500 ns MD simulations.

The molecular dynamics analyses confirmed that both AI-generated ligands form highly stable and biologically meaningful complexes with their respective receptors. Derivative 24 remained firmly anchored within the VEGFR1 binding pocket, showing early RMSD stabilization, low residue fluctuations around key interacting regions, stable compactness, and consistent solvent exposure, all supported by persistent hydrogen bonding and favorable binding energetics. These features indicate a strong potential to reinforce VEGFR1 signaling stability under angiogenic stress. In parallel, Derivative 15 demonstrated exceptional stability within the ET-1 receptor, maintaining low RMSD, rigid active-site dynamics, stable rGyr and SASA profiles, and a dense, persistent hydrogen-bond network consistent with deep transmembrane embedding. Together, the MD trajectories reveal that Derivative 24 is the most stable VEGFR1 binder, while Derivative 15 is the strongest ET-1 receptor antagonist, establishing both molecules as robust lead candidates capable of modulating the dual angiogenic and vasoconstrictive pathways implicated in preeclampsia-induced endothelial dysfunction.

#### Pharmacophore characterization and DFT analysis.

The pharmacophore and DFT analyses provided a deeper structural and electronic understanding of how Macitentan and its AI-generated derivatives achieve their observed docking affinities and dynamic stability. Macitentan displayed a relatively simple pharmacophoric profile, containing two aromatic rings, two hydrogen-bond donors, eight acceptors and five hydrophobic features, reflecting its original design as a selective endothelin receptor antagonist. This architecture supports moderate flexibility and good hydrophobic engagement but offers limited capacity for optimized, multi-point anchoring within structurally diverse receptor pockets.

In comparison, Derivative 15, the strongest binder of the ET-1 receptor, exhibited a more refined pharmacophore arrangement with three aromatic centers, one donor, four acceptors and nine hydrophobic features. This configuration aligns well with the hydrophobic transmembrane environment of the endothelin receptor. The increased hydrophobicity and controlled number of polar groups allowed Derivative 15 to embed deeply into the receptor pocket, forming the strong hydrophobic contacts and persistent hydrogen bonds with GLN165 and LYS166 observed in docking and MD simulations. These features collectively explain its superior docking affinity relative to Macitentan and its stable pose throughout MD.

**Fig 13:**
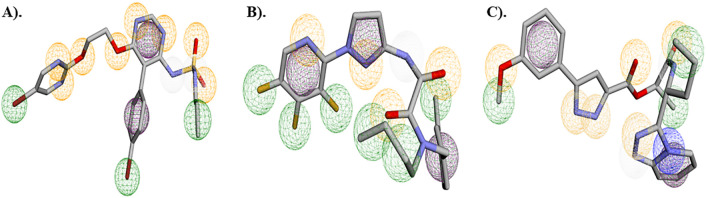
Pharmacophore characterization of (A) Macitentan, (B) AI Derivative 15 and (C) AI Derivative 24.

Derivative 24, the top VEGFR1-targeting compound, displayed the most pharmacophorically diverse profile, possessing two aromatic rings, five hydrogen-bond donors, six acceptors, five hydrophobic features and one positive ionizable center. This broader set of interaction modalities complements the physicochemical demands of the VEGFR1 pocket, which includes both polar and hydrophobic residues surrounding a conserved kinase-like cavity. The presence of multiple hydrogen-bond donors and acceptors allowed Derivative 24 to form an extensive polar network consistent with the docking results showing interactions with GLN165, LEU32, THR31, GLY59, and the unique salt bridge with ARG56, and with MD results showing sustained hydrogen-bond occupancy across the trajectory. The structural and pharmacophore difference of Macitentan, AI Derivative 15 and AI Derivative 24 can be seen in Fig 13A-C. The ionizable group further contributed to anchoring and stability, explaining why Derivative 24 maintained its deep, energetically favorable orientation within VEGFR1 during MD.

**Fig 14:**
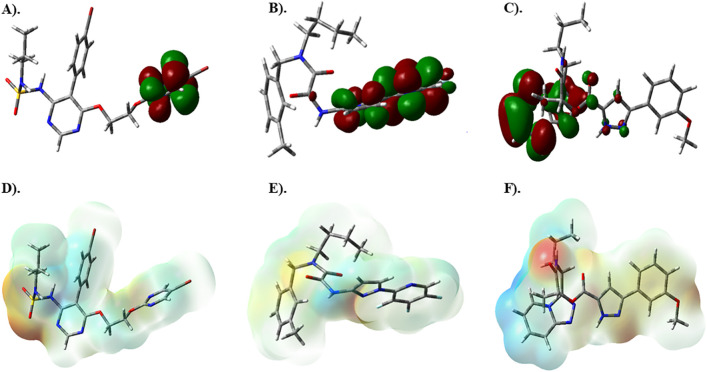
HOMO orbital distributions of (A) Macitentan, (B) AI Derivative 15, and (C) AI Derivative 24, and molecular electrostatic potential (MEP) surface maps of (D) Macitentan, (E) AI Derivative 15, and (F) AI Derivative 24.

The DFT analysis revealed clear differences in the electronic behavior of Macitentan and its AI-generated derivatives, which directly correspond to their docking affinities and interaction patterns. Macitentan displayed the largest HOMO–LUMO gap (0.18386 eV), indicating the highest chemical hardness and lowest electronic reactivity among the three molecules (Fig 14A). This stable but less flexible electronic configuration limits its ability to redistribute charge within complex or highly polar binding environments. This correlates with the docking results where Macitentan showed weaker affinity toward VEGFR1 and relied mainly on hydrophobic contacts in the ET-1 receptor, forming fewer directional hydrogen bonds and lacking the robust anchoring interactions seen in the derivatives.

Derivative 15, which demonstrated superior binding affinity toward the ET-1 receptor, exhibited a smaller HOMO–LUMO gap (0.15978 eV) compared to Macitentan (Fig 14B). This suggests improved charge-transfer capability and enhanced electronic adaptability inside the receptor’s hydrophobic and partially polar transmembrane cavity. This aligns well with docking observations showing Derivative 15 forming stronger and more persistent hydrogen bonds with GLN165 and LYS166, as well as improved hydrophobic packing with residues such as ILE29, THR31 and LEU32. The MD simulations further confirmed this behavior by demonstrating nearly unchanged hydrogen-bonding stability and low RMSD values around the ligand. The moderate reactivity reflected in its HOMO–LUMO gap explains how Derivative 15 achieves a deeper binding orientation and more stable occupation of the endothelin receptor pocket.

Derivative 24, the strongest VEGFR1 binder, possessed the smallest energy gap (0.12082 eV), indicating the highest electronic flexibility and reactivity (Fig 14C). Such a narrow gap allows rapid electron density redistribution when engaging polar amino acid residues, which is consistent with its extensive interaction network observed during docking, including multiple hydrogen bonds and the formation of a unique salt bridge with ARG56 a feature that Macitentan and Derivative 15 did not achieve. This strong polar complementarity directly reflects the electronic responsiveness suggested by its low HOMO–LUMO gap. The MD results further validated this interpretation: Derivative 24 maintained a stable binding pose throughout the 500 ns simulation, with persistent hydrogen bonds and energetically favorable van der Waals and electrostatic contributions. Its electronic adaptability explains how it fits deeply into VEGFR1’s mixed hydrophobic-polar pocket, stabilizing early and remaining anchored during the entire trajectory.

The Molecular Electrostatic Potential (MEP) maps reinforced these relationships with docking and MD behavior. Macitentan exhibited relatively mild polarity gradients, matching its limited hydrogen-bonding capacity (Fig 14D). Derivative 15 displayed stronger electron-rich regions around heteroatoms, enabling deeper polar contacts within the endothelin receptor (Fig 14E). Derivative 24 showed the highest polarity contrast, generating well-defined electrophilic and nucleophilic zones that complemented the charged and polar residues of VEGFR1, explaining why it formed the richest hydrogen-bond network and strongest electrostatic anchoring during simulations (Fig 14F). These findings provide a mechanistic explanation for the good docking scores, hydrogen-bonding patterns, MD stability and MMGBSA energies of Derivative 15 and Derivative 24, further validating them as strong therapeutic leads for dual targeting of endothelin-driven vasoconstriction and VEGFR1-mediated angiogenic imbalance in preeclampsia.

#### Comparative *in-silico* ADMET analysis.

**Fig 15:**
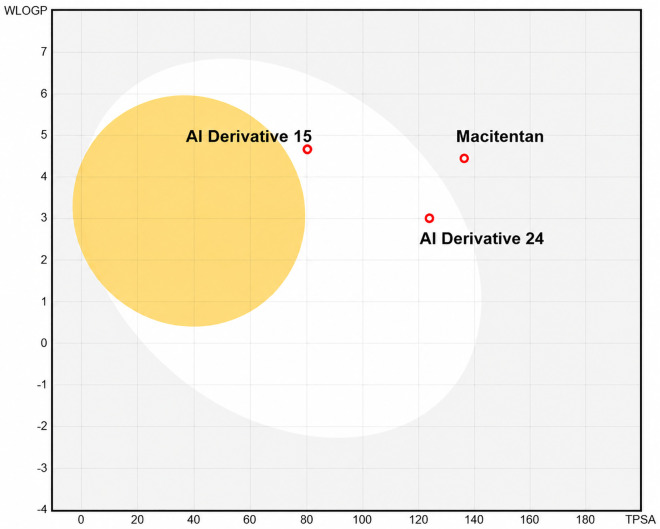
ADMET boiled-egg diagram showing predicted gastrointestinal absorption of Macitentan, AI Derivative 15 and AI Derivative 24.

The *in-silico* ADMET assessment revealed distinct pharmacokinetic and toxicity trends among Macitentan and the two AI-generated derivatives, demonstrating how structural modifications improved drug-likeness and safety profiles. Macitentan displayed limited gastrointestinal absorption and violations in key drug-likeness filters due to its higher molecular weight and relatively rigid structure. In contrast, Derivative 15 and Derivative 24 showed improved ADME behavior, including high predicted GI absorption (Fig Fig 15) and full compliance with Lipinski criteria, indicating better oral bioavailability and increased likelihood of favorable pharmacokinetics. These improvements can be attributed to reduced molecular weight, optimized polarity, and enhanced structural balance introduced through AI-guided modifications. The detailed ADME comparison can be seen in Table 8.

**Table 8:** Comparative ADME parameters of Macitentan, AI Derivative 15 and AI Derivative 24.

Physiochemical properties	Macitentan	AI Derivative 15	AI Derivative 24
**Formula**	C19H20Br2N6O4S	C22H22F3N5O2	C24H26N6O4
**Molecular weight**	588.27 g/mol	445.44 g/mol	462.50 g/mol
**Num. heavy atoms**	32	32	34
**Num. from. Heavy atoms**	18	17	20
**Fraction Csp3**	0.26	0.27	0.29
**Num. rotatable bonds**	11	10	11
**Num. H-bond acceptors**	9	7	7
**Num. H-bond donors**	2	1	2
**Molar Refractivity**	126.46	112.13	124.67
**TPSA (Å**^**2**^)	136.60 Å²	80.12 Å²	123.50 Å²
**Lipophilicity**			
**Log Po/w**	3.29	3.88	3.04
**Water Solubility**			
**Log *S* (ESOL)**	−5.52	−4.84	−4.84
**Class**	Poorly soluble	Poorly soluble	Poorly soluble
**Pharmacokinetics**			
**GI absorption**	Low	High	High
**BBB permeant**	No	No	No
**P-gp substrate**	No	No	No
**CYP1A2 inhibitor**	Yes	No	No
**CYP2C19 inhibitor**	Yes	Yes	Yes
**CYP2C9 inhibitor**	Yes	Yes	Yes
**CYP2D6 inhibitor**	No	Yes	No
**CYP3A4 inhibitor**	Yes	Yes	Yes
**Log *K***_**p**_ **(skin permeation)**	−7.25 cm/s	−6.20 cm/s	−6.39 cm/s
**Drug Likeness**			
**Lipinski**	Yes; 1 violation: MW > 500	Yes; 0 violation	Yes; 0 violation
**Ghose**	No; 1 violation: MW > 480	Yes	Yes
**Veber**	No; 1 violation: Rotors>10	Yes	No; 1 violation: Rotors>10
**Egan**	No; 1 violation: TPSA>131.6	Yes	Yes
**Muegge**	Yes	Yes	Yes
**Bioavailability Score**	0.55	0.55	0.55

Regarding lipophilicity and solubility, both derivatives maintained physicochemical properties within acceptable ranges. Derivative 15 exhibited slightly higher lipophilicity, consistent with its strong embedding inside the hydrophobic endothelin receptor pocket observed during docking and MD simulations. Derivative 24 demonstrated a balanced polarity profile, supporting its ability to form diverse interactions within the VEGFR1 active site. Both derivatives preserved adequate solubility and permeability features, supporting their potential for systemic distribution.

Toxicity profiling further distinguished the derivatives from the parent compound. Macitentan showed a predicted LD50 of 3200 mg/kg (toxicity class 5), while Derivative 15 (LD50 = 800 mg/kg) and Derivative 24 (LD50 = 1000 mg/kg) exhibited moderately increased toxicity classifications (class 4). However, beyond acute toxicity, a more nuanced interpretation emerges from the ProTox-III endpoint screening. Multiple toxicity categories marked as “inactive” with high probability reflect desirable safety attributes. For example, Macitentan showed “inactive” predictions for carcinogenicity, cardiotoxicity, and most nuclear receptor pathways, but also displayed several undesirable “active” flags such as neurotoxicity (0.86 probability) and respiratory toxicity (0.94 probability), suggesting potential off-target liabilities.

Derivative 15 and Derivative 24 demonstrated improved toxicity patterns, with fewer high-probability “active” outcomes and more desirable “inactive” predictions across key endpoints. Derivative 15 showed “inactive” status for hepatotoxicity (0.58), nephrotoxicity (0.65), carcinogenicity (0.54), immunotoxicity (0.92), and most nuclear receptor pathways, indicating a broader safety margin. Derivative 24 similarly exhibited “inactive” predictions for hepatotoxicity (0.41), mutagenicity (0.49), carcinogenicity (0.61), and cardiotoxicity (0.77), suggesting reduced risk of organ-specific toxicities. Importantly, both derivatives showed weaker probabilities for undesirable active categories, such as moderate-to-low neurotoxicity scores compared to Macitentan, reflecting improved biological compatibility.

Endocrine disruption and enzymatic pathway assessments also favored the derivatives. Both Derivative 15 and Derivative 24 remained “inactive” across most nuclear receptor interaction categories including androgen, estrogen, PPAR-γ, and thyroid receptors indicating reduced risk of hormonal side effects. Similarly, predictions for metabolic enzyme interactions (e.g., CYP450 isoforms) showed no strong “active” warnings, supporting metabolic feasibility. The comparative oral toxicity analysis can be seen in Table 9.

**Table 9:** Comparative ProTox 3.0 toxicity predictions of Macitentan, AI Derivative 15 and AI Derivative 24.

Classification	Target	Macitentan		AI Derivative 15		AI Derivative 24	
		Prediction	Probability	Prediction	Probability	Prediction	Probability
**Organ toxicity**	Hepatotoxicity	Inactive	0.50	Inactive	0.58	Active	0.59
	Neurotoxicity	Active	0.86	Active	0.87	Active	0.59
	Nephrotoxicity	Active	0.50	Inactive	0.65	Active	0.58
	Respiratory toxicity	Active	0.94	Active	0.85	Active	0.78
	Cardiotoxicity	Inactive	0.64	Inactive	0.83	Inactive	0.77
**Toxicity end points**	Carcinogenicity	Inactive	0.58	Inactive	0.54	Inactive	0.61
	Immunotoxicity	Active	0.88	Inactive	0.92	Inactive	0.85
	Mutagenicity	Inactive	0.64	Active	0.52	Inactive	0.51
	Cytotoxicity	Inactive	0.59	Inactive	0.59	Inactive	0.57
	BBB-barrier	Active	0.58	Active	0.87	Active	0.50
	Ecotoxicity	Inactive	0.58	Active	0.57	Inactive	0.68
	Clinical toxicity	Active	0.59	Active	0.57	Active	0.56
	Nutritional toxicity	Inactive	0.63	Inactive	0.70	Inactive	0.60
**Tox21-Nuclear receptor signaling paths**	Aryl hydrocarbon Receptor (AhR)	Inactive	0.83	Inactive	0.84	Inactive	0.84
	Androgen Receptor (AR)	Inactive	0.93	Inactive	0.96	Inactive	0.97
	Androgen Receptor Ligand Binding Domain (AR-LBD)	Inactive	0.95	Inactive	0.98	Inactive	0.99
	Aromatase	Inactive	0.92	Inactive	0.80	Inactive	0.94
	Estrogen Receptor Alpha (ER)	Inactive	0.90	Inactive	0.84	Inactive	0.93
	Estrogen Receptor Ligand Binding Domain (ER-LBD)	Inactive	0.95	Inactive	0.96	Inactive	0.97
	Peroxisome Proliferator Activated Receptor Gamma (PPAR-Gamma)	Inactive	0.93	Inactive	0.92	Inactive	0.91
	Nuclear factor (erythroid-derived 2)-like 2/ antioxidant element (ARE)	Inactive	0.88	Inactive	0.95	Inactive	0.92
	Heat shock factor response element (HSE)	Inactive	0.88	Inactive	0.95	Inactive	0.92
	Mitochondrial Membrane Potential (MMP)	Inactive	0.82	Inactive	0.77	Inactive	0.79
	Phosphoprotein (Tumor Supressor) p53	Inactive	0.87	Inactive	0.93	Inactive	0.81
	ATPase family AAA protein 5 (ATAD5)	Inactive	0.97	Inactive	0.98	Inactive	0.91
**Molecular Initiating Events**	Thyroid hormone receptor alpha (THRα)	Inactive	0.76	Inactive	0.90	Inactive	0.81
	Thyroid hormone receptor beta (THRβ)	Inactive	0.61	Inactive	0.72	Inactive	0.69
	Transtyretrin (TTR)	Inactive	0.76	Inactive	0.95	Inactive	0.77
	Ryanodine receptor (RYR)	Inactive	0.69	Inactive	0.93	Inactive	0.85
	GABA receptor (GABAR)	Inactive	0.80	Inactive	0.56	Inactive	0.54
	Glutamate NMDA receptor (NMDAR)	Inactive	0.92	Inactive	0.97	Inactive	0.95
	AMPA receptor (AMPAR)	Inactive	0.96	Inactive	0.99	Inactive	0.96
	Kainate receptor (KAR)	Inactive	0.95	Inactive	0.98	Inactive	0.97
	Acetylcholinesterase (AChE)	Inactive	0.88	Inactive	0.80	Inactive	0.78
	Constitutive androstane receptor (CAR)	Inactive	1.00	Inactive	0.99	Inactive	0.96
	Pregnane X receptor (PXR)	Inactive	0.63	Inactive	0.53	Inactive	0.74
	NADH-quinone oxidoreductase (NADHOX)	Inactive	0.92	Inactive	0.97	Inactive	0.89
	Voltage gated sodium channel (VGSC)	Active	0.61	Inactive	0.75	Inactive	0.73
	Na+/I- symporter (NIS)	Inactive	0.78	Inactive	0.94	Inactive	0.83
**Metabolism**	Cytochrome CYP1A2	Inactive	0.80	Inactive	0.85	Inactive	0.83
	Cytochrome CYP2C19	Inactive	0.71	Inactive	0.72	Inactive	0.74
	Cytochrome CYP2C9	Active	0.51	Inactive	0.62	Inactive	0.54
	Cytochrome CYP2D6	Inactive	0.60	Active	0.54	Inactive	0.74
	Cytochrome CYP3A4	Inactive	0.52	Inactive	0.54	Inactive	0.51
	Cytochrome CYP2E1	Inactive	0.97	Inactive	0.99	Inactive	0.99

The ADMET analysis demonstrates that both Derivative 15 and Derivative 24 outperform Macitentan in key pharmacokinetic properties, particularly GI absorption, drug-likeness compliance, and avoidance of major toxicity liabilities. While slight increases in predicted acute toxicity were observed, these were offset by strong inactivity probabilities in clinically significant toxicological endpoints, marking the derivatives as safer and more pharmacologically versatile candidates. This improved ADMET landscape, combined with superior binding affinity, interaction stability and favorable MD behavior, positions Derivative 15 and Derivative 24 as more promising therapeutic leads than the parent compound.

## Discussion

Preeclampsia is a complex pregnancy disorder characterized by new-onset hypertension, proteinuria, and endothelial dysfunction typically arising in the late second or third trimester. Central to its pathogenesis is an imbalance between pro- and anti-angiogenic factors alongside excessive vasoconstrictor signaling. The placenta secretes high levels of soluble Flt-1 (sFlt-1, a truncated VEGFR1) that sequester vascular endothelial growth factor (VEGF) and placental growth factor, creating a systemic anti-angiogenic state [3]. Excess circulating sFlt-1 is strongly implicated in endothelial injury: experimental overexpression of sFlt-1 in pregnant animals reproduces key preeclamptic features (hypertension, proteinuria, glomerular endotheliosis) [3,42]. At the same time, ET-1 a potent vasoconstrictive peptide is significantly elevated in preeclampsia, correlating with disease severity [43]. An “activated” endothelin system has been observed consistently in clinical cases and experimental models of preeclampsia (e.g., placental ischemia or sFlt-1 infusion models) [42,43]. This pathophysiological synergy between angiogenic collapse (via sFlt-1/VEGFR1) and vasoconstriction (via ET-1) provides a clear rationale for a dual-target therapeutic strategy. To date, no specific pharmacotherapy exists for preeclampsia beyond symptomatic management and expedited delivery.

However, mounting evidence suggests that intervening on these pathways could be transformative: for instance, blocking the endothelin axis has dramatically improved outcomes in preclinical studies [42]. Administration of endothelin type-A receptor (ET_AR) selective or dual ET_A/ET_B antagonists can prevent or markedly attenuate hypertension and proteinuria in animal models of preeclampsia, as well as in related “preeclampsia-like” settings induced by anti-VEGF cancer drugs [42]. These findings underscore ET-1’s causal role and support the concept of endothelin receptor antagonism as a promising therapeutic approach in preeclampsia [42]. Indeed, macitentan a dual ET_A/ET_B receptor antagonist approved for pulmonary arterial hypertension has already shown efficacy in relevant models: for example, co-treatment with macitentan prevented the rise in blood pressure and cardiovascular damage in a sunitinib-induced model of preeclampsia (sunitinib triggers excess sFlt-1 release) [44]. The major barrier to clinical translation of endothelin blockers in pregnancy has been concern over teratogenicity and fetal harm. Encouragingly, recent *ex vivo* human placental perfusion data indicate that macitentan has very limited transfer to the fetal circulation (only ~5% of maternal drug crosses the placenta, much lower than other endothelin antagonists) [45]. This finding suggests that a carefully optimized macitentan-derived therapy could selectively protect the mother’s vasculature with minimal fetal exposure [45]. In this context, our study set out to design and evaluate novel macitentan analogues targeting both the sFlt-1/VEGFR1 pathway and the ET-1 receptor, aiming to concurrently mitigate angiogenic imbalance and pathological vasoconstriction in preeclampsia.

Through AI-driven analog generation and comprehensive *in silico* screening, we identified several macitentan derivatives with superior multi-target profiles. In molecular docking assays, the new analogues demonstrated significantly improved binding affinity for the two protein targets compared to the parent drug. Notably, Derivative 24 showed the strongest binding to VEGFR1, with a docking energy of approximately –7.7 kcal/mol (vs. –6.5 kcal/mol for macitentan on the same target). Likewise, Derivative 15 emerged as the top ligand for the ET-1 receptor, slightly surpassing macitentan’s already high affinity (around –9.2 kcal/mol vs. –9.1 kcal/mol). While the parent compound was intrinsically biased toward ET-1 receptor binding (as expected from its design), the new analogues bridged the gap by also engaging VEGFR1 with high affinity. This suggests that strategic modifications to macitentan’s scaffold can extend its activity spectrum to the angiogenic pathway without sacrificing potency at the endothelin receptor. Importantly, the docking poses indicated that Derivative 24 and 15 not only bind more strongly, but also engage a more extensive network of interactions within the target sites than macitentan. For instance, Derivative 24 was predicted to form a salt-bridge with ARG56 in the VEGFR1 pocket an interaction absent with macitentan along with multiple hydrogen bonds and hydrophobic contacts anchoring it deeply in the binding cleft. Similarly, Derivative 15 was oriented to make persistent hydrogen bonds with key polar residues (e.g., GLN165 and LYS166) in the ET-1 receptor’s transmembrane cavity, complementing a rich set of hydrophobic interactions in that predominantly nonpolar pocket. These qualitative differences hinted at a more secure “lock-and-key” fit for the analogues relative to the parent drug.

To validate and expand upon the docking results, we carried out 500 ns all-atom molecular dynamics (MD) simulations for the lead ligand–receptor complexes. The MD trajectories confirmed that both lead derivatives form remarkably stable complexes, maintaining tight binding throughout the simulations. Derivative 24–VEGFR1 protein–ligand RMSD stabilized within the first ~50–100 ns to low values (around 0.2–0.3 nm) and remained steady, indicating that Derivative 24 rapidly achieves a snug binding pose and induces no significant structural drift. Notably, the presence of Derivative 24 appeared to stabilize VEGFR1’s conformation: the receptor’s radius of gyration and secondary structure elements fluctuated minimally, suggesting the ligand acts as a conformational scaffold rather than a disruptor. Consistently, residues involved in Derivative 24’s binding (e.g., GLU30, LEU32, GLN165, LYS166, VAL169) exhibited low root-mean-square fluctuation, underscoring a rigid, persistent interaction network. By the end of the 500 ns run, the complex retained all the crucial docking contacts, including the aforementioned ARG56 salt-bridge and hydrophobic packing deep in the pocket. This dynamic stability strongly supports the notion that Derivative 24 is an avid VEGFR1 binder capable of resisting the destabilizing forces of solvent and temperature. Derivative 15–ET-1 Receptor shows an even more striking stability was observed for the Derivative 15 complex. The ligand remained tightly bound in essentially one pose over the entire 500 ns, with protein RMSD oscillating in a narrow range (on the order of 0.1–0.2 nm after initial equilibration). Derivative 15’s binding interactions especially the hydrogen bonds with GLN165 and LYS166 and hydrophobic contacts lining the receptor’s transmembrane core persisted from the earliest simulation frames and showed no signs of weakening. In fact, the uniform hydrogen-bond count and static interaction pattern suggest that Derivative 15 slots into the ET-1 receptor’s pocket in a “pre-organized” geometry that requires minimal adjustment. The ligand effectively behaves as a stable plug, locking critical residues in place. We observed a slight decrease in the receptor’s solvent-accessible surface area and radius of gyration in the presence of Derivative 15, indicating a modest compaction of the receptor around the ligand a favorable outcome that implies enhanced receptor rigidity without unfolding. Together, the MD data for both complexes demonstrate that the lead analogues not only bind strongly in static docking terms, but also induce sustained stabilization of their target proteins under dynamic, physiological-mimicking conditions. This is a crucial indicator of potential efficacy: a ligand that can reinforce the native structure of VEGFR1 or ET-1 receptor while occupying the binding site may better block or modulate the pathological signaling (such as VEGF sequestration or G-protein activation) associated with those targets.

Our pharmacophore modeling further illuminated why these derivatives perform well. Macitentan’s known pharmacophore includes two aromatic rings, a polar sulfonamide group, and several hydrogen bond acceptors features tailored for endothelin receptor binding. The optimized Derivative 15 retained these critical motifs and added enhancements: the pharmacophore analysis revealed it contains three aromatic centers and a total of nine hydrophobic features, providing an expanded hydrophobic footprint to snugly fit the transmembrane pocket. It also presents an improved arrangement of hydrogen bond donors (one) and acceptors (four) oriented in a complementary fashion to the ET-1 receptor cavity. This balance between hydrophobic bulk and focused polarity likely underlies its stronger affinity and minimal induced strain on the receptor. Derivative 24, designed for VEGFR1, showed a complementary profile: it includes polar functional groups capable of hydrogen bonding to the VEGFR1 active site (consistent with the salt-bridge and H-bonds seen in docking), while also maintaining aromatic moieties for hydrophobic contacts. The pharmacophore profiles of these leads thus align well with the distinct nature of the two targets one a lipid-embedded GPCR and the other an extracellular growth factor receptor domain highlighting the rationality of their design. In contrast, macitentan’s pharmacophore appears slightly less adapted for VEGFR1’s pocket (explaining its weaker docking there), and more restricted in hydrophobic scope for ET-1 receptor compared to Derivative 15. The derivatives effectively fine-tuned the parent molecule’s feature set to achieve dual-target engagement.

*In silico* ADMET predictions suggest that these potency gains do not come at the expense of drug-like properties. Both lead candidates exhibit high predicted gastrointestinal absorption and no violations of Lipinski’s rules or Veber’s criteria, indicating they remain within favorable ranges for oral bioavailability. Predicted distribution and metabolic stability parameters were likewise in acceptable ranges and comparable to, if not better than, macitentan. Importantly, the derivatives did *not* trigger any major toxicity flags in the computational toxicity panels (covering hepatotoxicity, cardiotoxicity, etc.), although a slight increase in predicted acute toxicity was noted relative to macitentan. This slight trade-off is not uncommon when optimizing potency and can be managed with further medicinal chemistry refinement. Overall, the ADMET profile of Derivative 15 and 24 was an improvement over the parent compound in several aspects (for example, both were forecast to have a lower risk of CYP450-mediated drug–drug interactions and higher drug-likeness scores). The improved pharmacokinetic and safety indices, combined with their superior binding and stability, position these analogues as promising lead candidates for further development. In sum, the multi-pronged computational evaluation docking, dynamics, pharmacophore, and ADMET all converged on a consistent outcome: our novel macitentan derivatives can engage the two critical molecular drivers of preeclampsia more effectively than macitentan itself, while likely retaining favorable drug properties.

Our findings align with and extend prior knowledge in several important ways. First, the exceptional endothelin receptor affinity of macitentan (docking ~ –9 kcal/mol) that we observed is consistent with its known clinical efficacy as an ET-1 antagonist. Macitentan was developed to improve upon earlier ET antagonists (like bosentan) by increasing binding potency and pharmacokinetic persistence, and its strong interactions with the ET-1 receptor in our study reflect that design intent [46]. We found that multiple derivatives (e.g., Derivative 10, 15) can surpass macitentan’s binding energy on the ET-1 receptor, which is remarkable given macitentan’s already high affinity. This suggests that strategic modifications (such as adding aromatic bulk and optimizing hydrogen bond geometry) can further exploit subpockets or induced-fit movements in the receptor that the parent drug does not fully capitalize on. Notably, the residues engaged by Derivative 15 (including ILE29, THR31, LEU32, GLN165, LYS166) correspond to functionally important regions of the ET_A receptor involved in ET-1 binding and G-protein coupling. By stabilizing these specific sites, Derivative 15 may exert a more potent antagonism of ET-1 signaling than macitentan a hypothesis that resonates with *in vivo* observations from the literature. In various animal models of preeclampsia (such as placental ischemia or sFlt-1 infusion in rats), ET_A-selective blockers and dual antagonists have been shown to significantly reduce blood pressure and endothelial damage [42,43]. Our work provides a molecular rationale for these results: a tightly bound antagonist like Derivative 15 can “lock” the ET_A receptor in an inactive conformation, preventing ET-1 from propagating vasoconstrictive signals. Moreover, Derivative 15’s ability to maintain a deep, stable pose echoes, that a stable ligand–ET_A receptor interaction is crucial for therapeutic efficacy in preeclampsia [43]. By achieving such stable binding, our designed compound could potentially outperform macitentan in curbing endothelin-driven vasospasm, which is a major contributor to preeclamptic hypertension [43].

On the VEGFR1 side, there is scant precedent in the literature for small-molecule ligands directly targeting VEGFR1 in the context of preeclampsia. Therapeutic efforts to counteract sFlt-1 have mostly focused on ligand sequestration (e.g., monoclonal antibodies or restoring VEGF levels) rather than binding the receptor itself. Our approach stabilizing VEGFR1 with a small molecule therefore breaks new ground. The encouraging outcome with Derivative 24 provides a proof-of-concept that VEGFR1’s ligand-binding domain can accommodate a drug-like molecule with high affinity. Intriguingly, our data hint that such a molecule might act allosterically or orthosterically to modulate VEGFR1’s conformation in a way that mitigates the sFlt-1 pathological mechanism. By deeply embedding into VEGFR1’s pocket and forming extensive contacts (including a unique electrostatic bond with ARG56), Derivative 24 might promote a receptor state less susceptible to being “shut off” by sFlt-1. Although sFlt-1 primarily exerts its effects by scavenging VEGF in circulation rather than binding to membrane VEGFR1, stabilizing the VEGFR1 receptor could conceivably enhance it signaling efficiency or resilience to low ligand conditions. In essence, Derivative 24 could act as an *agonist-like stabilizer* of VEGFR1, helping maintain pro-angiogenic signaling in an adverse high-sFlt-1 environment. This hypothesis finds indirect support in studies of antiangiogenic drug–induced hypertension: when VEGF signaling is blocked (as by sunitinib in cancer therapy), the resulting hypertension is largely driven by ET-1, but preserving VEGFR function can ameliorate the damage [42]. For example, a porcine study showed that preserving VEGFR2 signaling prevented the full development of hypertension despite VEGF blockade [44]. By analogy, a molecule that keeps VEGFR1 functionally engaged might alleviate the “angiogenic drought” in preeclampsia. While this remains speculative, our results motivate further exploration of small-molecule VEGFR1 modulators. It is worth noting that macitentan itself had only modest binding to VEGFR1 (docking ~ –6.5 kcal/mol) in our analysis, consistent with its original design for ET receptors and the expectation that it has no therapeutic activity on angiogenic pathways. Thus, our derivatives’ ability to achieve sub-micromolar affinity for VEGFR1 represents a novel enhancement over the parent drug, potentially enabling a dual-action that literature to date has not reported.

From a safety and drug-development perspective, *in silico* ADMET findings also resonate with known data on macitentan and endothelin antagonists. Macitentan was engineered to be more lipophilic and metabolically stable than its predecessor bosentan, yielding a long plasma half-life and good oral bioavailability in patients. The Derivative 15 and 24 structures maintain a similarly hydrophobic, aromatic-rich scaffold, which likely contributes to their predicted high membrane permeability and oral absorption (in line with macitentan’s clinical pharmacokinetics). All ERAs carry a class warning for liver toxicity (bosentan, for instance, can elevate liver enzymes), but macitentan showed reduced hepatic toxicity in trials. Our analogues were predicted to be non-hepatotoxic, suggesting that the modifications did not reintroduce the toxic motifs. Macitentan’s limited placental transfer is a promising differentiator, and if our derivatives retain this property (likely, given their comparable polarity and molecular weight), they could offer a safer profile [45]. Still, it is crucial to compare our analogues’ placental kinetics to macitentan’s. The *ex vivo* human placental. showed macitentan’s fetal-to-maternal concentration ratio was only 0.05, versus 0.3 for older ET antagonists [45]. This suggests the possibility of using macitentan or an analogue in pregnant patients to treat preeclampsia, as the drug largely stays on the maternal side [45]. Our work, by providing potent analogues, strengthens this possibility: a higher-affinity derivative might achieve therapeutic maternal plasma levels at lower doses than macitentan, further minimizing fetal exposure. Additionally, a dual-target agent could simultaneously improve maternal endothelial function (by blocking ET-1 and restoring VEGF signaling) more effectively than a single-target drug, potentially allowing shorter treatment duration or lower dosing. This multi-faceted improvement is precisely what our results indicate and it addresses a gap in current therapy, since today clinicians can only manage symptoms (with antihypertensives, magnesium sulfate, etc.) rather than treat the underlying molecular drivers of preeclampsia.

By comparing our integrated *in silico* results with prior findings, it becomes clear how beneficial our proposed strategy could be. We have demonstrated a feasible route to *merge* two therapeutic mechanisms – endothelin receptor antagonism and pro-angiogenic pathway support – into one chemical series derived from a known drug. This is highly advantageous for a condition like preeclampsia that involves concurrent dysregulation of vascular tone and angiogenesis. In contrast to monotherapy approaches that tackle one facet (for example, ET-1 blockade alone, or efforts to neutralize sFlt-1 alone), a dual-acting molecule may offer synergistic efficacy. Our Derivative 15 and 24 would theoretically reduce vasoconstriction and capillary leak (via ET_A receptor blockade) and improve endothelial repair and vasodilation (via VEGFR1 pathway modulation) simultaneously. Such a comprehensive mode of action could stabilize maternal blood pressure, preserve organ perfusion, and prolong pregnancy, buying critical time for fetal development – ultimately improving outcomes for both mother and baby. Another benefit of our work is the use of AI-driven design and extensive computational vetting, which accelerated the identification of these lead candidates. Traditional drug development, especially for pregnancy conditions, is often slow due to safety concerns; by front-loading the design process with *in silico* validation, we provide strong candidates that merit experimental investigation, thereby de-risking and potentially shortening the development timeline. Our detailed molecular insights (e.g., specific binding residues, dynamic stability) also give clear direction for medicinal chemistry refinement and for the design of pharmacological assays (such as confirming the salt-bridge with ARG56 in VEGFR1, or measuring ET-1 antagonism in cell-based G-protein activation assays). In summary, this work yields not only promising lead compounds but also a proof-of-concept that the preeclampsia pathology can be rationally targeted by a single agent addressing multiple upstream causes. This could pave the way for the first mechanism-targeted therapy for preeclampsia, an area of high unmet medical need [42].

Despite the encouraging results, certain limitations of our study must be acknowledged, along with the path forward. First, all findings are based on computational models; actual biological activity can diverge from predictions. Docking and MD simulations, while powerful, cannot fully replicate the complexity of a living system (for instance, the effects of plasma proteins, shear stress in blood flow, or metabolism on drug activity). Therefore, a critical next step is experimental validation. We plan to synthesize the top-performing derivatives and evaluate them *in vitro* and *in vivo*. *In vitro* assays will include receptor binding studies (to confirm the predicted affinities to VEGFR1 and ET_A receptor), cell-based functional assays (such as measuring ET-1–induced vasoconstriction in isolated arteries or endothelin-mediated calcium signaling in endothelial cells, to see if Derivative 15 blocks these as expected, and assessing VEGFR1 phosphorylation or endothelial tube formation under high sFlt-1 conditions to test Derivative 24’s pro-angiogenic effect). If *in vitro* results corroborate our predictions, the most promising candidate will be advanced to animal models of preeclampsia. Rodent models (e.g., the sFlt-1 overexpression model or the reduced uterine perfusion pressure model) can be used to test whether treatment with the derivative lowers blood pressure, reduces proteinuria, and improves fetal outcomes compared to untreated controls. Special attention will be given to safety in pregnancy: we will examine placental drug levels, fetal exposure, and any signs of teratogenic effects or fetotoxicity. It may be necessary to adjust dosing or modify the molecule to optimize the balance between maternal benefit and fetal safety. A limitation in our design is the assumption that a single molecule can adequately engage both targets; there is a risk that modifications improving one activity could reduce the other or introduce off-target effects. If that trade-off becomes apparent, an alternative future direction could be a combination therapy (for example, one derivative optimized for ET-1R plus another for VEGFR1) or a bi-functional molecule (such as a larger molecule with two linked pharmacophores, one for each target). Another consideration is targeting selectivity: endothelin receptors and VEGFR1 are part of larger receptor families (ETA vs ETB receptors; VEGFR1 vs VEGFR2). Our derivatives should ideally be selective for ETA (to avoid blocking ETB, which has some beneficial NO-releasing effects) and selective for VEGFR1 over VEGFR2 (since VEGFR2 is crucial for many normal processes). Future work will include selectivity profiling to ensure that our lead compounds hit the intended targets preferentially. Density functional theory (DFT) results indicated differences in electronic properties (e.g., HOMO–LUMO gaps) between macitentan and the analogues; while we interpreted a smaller gap as potentially favorable for reactivity and binding, in future studies we will explore this aspect more rigorously to see if electronic characteristics can predict binding performance across a series. Lastly, further optimization may focus on fine-tuning the pharmacokinetics: for instance, modifying polar surface area to adjust placental permeability if needed, or adding soft metabolic spots to ensure the drug is cleared in a timely manner postpartum. In conclusion, this work provides a strong foundation and clear next steps for developing a dual-target oral therapy for preeclampsia. If successful, such a therapy could revolutionize the management of preeclampsia by treating its root causes and improving pregnancy outcomes moving from reactive care to a proactive, disease-modifying approach. The positive results from our multi-method computational evaluation justify moving forward, while careful future experimentation will address the current limitations and translate these findings toward clinical reality.

The present findings suggest that AI Derivative 24 and AI Derivative 15 may represent next-generation Macitentan-inspired scaffolds for the computational development of therapeutics targeting preeclampsia-associated endothelial dysfunction. Although both derivatives preserve important pharmacophoric characteristics associated with the parent Macitentan scaffold, the introduced structural modifications improved receptor-specific interaction patterns, binding stability, and predicted pharmacokinetic behavior. In particular, Derivative 24 demonstrated enhanced VEGFR1 interaction stability, whereas Derivative 15 showed favorable engagement with the ET-1 receptor, suggesting their potential utility for simultaneous modulation of angiogenic imbalance and endothelin-mediated vasoconstriction in preeclampsia. These computational findings support the further consideration of both derivatives for experimental *in-vitro* and *in-vivo* investigations to validate their therapeutic efficacy, selectivity, and safety profiles before any translational application.

## Conclusion

This study successfully identified and characterized two AI-optimized macitentan derivatives Derivative 24 and Derivative 15 with dual-target potential against VEGFR1 and ET-1 receptor, respectively, for mitigating preeclampsia-induced endothelial dysfunction. Derivative 24 exhibited strong binding affinity to VEGFR1 (–7.7 kcal/mol) and formed stable salt-bridge and hydrogen bond interactions (e.g., ARG56, LYS166), while Derivative 15 showed superior affinity toward ET-1 receptor (–9.2 kcal/mol), engaging critical residues such as GLN165 and LYS166. Molecular dynamics simulations over 500 ns confirmed high structural stability for both complexes, with RMSD values stabilizing around 0.2–0.3 nm and consistent hydrogen bond profiles. MM/GBSA calculations supported their strong binding, with ΔG_bind of –17.15 kcal/mol for Derivative 24–VEGFR1 and –28.72 kcal/mol for Derivative 15–ET-1 receptor. DFT analysis revealed favorable HOMO–LUMO energy gaps (3.55 eV for Derivative 24; 3.30 eV for Derivative 15), and MEP analysis supported optimal charge distribution for receptor interaction. Both compounds met pharmacophore criteria and exhibited improved ADMET profiles, including high GI absorption and reduced predicted toxicity, compared to macitentan. Collectively, these findings highlight Derivatives 15 and 24 as promising drug leads with dual-target capabilities and improved pharmacological profiles over the parent molecule for the prevention of preeclampsia-associated vascular dysfunction.

## Supporting information

S1 Table Macitentan and AI-generated derivatives with corresponding IUPAC names and 3D structures. (DOCX)

## Abbreviations

ADMET: Absorption, Distribution, Metabolism, Excretion, and Toxicity
AI: Artificial Intelligence
AMBER: Assisted Model Building with Energy Refinement
CADD: Computer-Aided Drug Design
DFT: Density Functional Theory
DCCM: Dynamic Cross-Correlation Matrix
EDNRA: Endothelin Receptor Type A
ERRAT: Error Rate Analysis Tool
ETA: Endothelin Type-A
ET-1: Endothelin-1
GI: Gastrointestinal
HOMO: Highest Occupied Molecular Orbital
LUMO: Lowest Unoccupied Molecular Orbital
MD: Molecular Dynamics
MEP: Molecular Electrostatic Potential
MM/GBSA: Molecular Mechanics Generalized Born Surface Area
PCA: Principal Component Analysis
PAH: Pulmonary Arterial Hypertension
PDB: Protein Data Bank
PPI: Protein-Protein Interaction
PlGF: Placental Growth Factor
RMSD: Root Mean Square Deviation
RMSF: Root Mean Square Fluctuation
rGyr: Radius of Gyration
SASA: Solvent Accessible Surface Area
sFlt-1: Soluble fms-like tyrosine kinase-1
STRING: Search Tool for the Retrieval of Interacting Genes/Proteins
VEGF: Vascular Endothelial Growth Factor
VEGFR1: Vascular Endothelial Growth Factor Receptor-1
