# Supplementary material for: AI-driven molecular diversification and ligand-based optimization of macitentan derivatives targeting VEGFR1 and endothelin signaling pathways
Source: PLoS One. 2026 Jun 30;21(6):e0352451. doi: 10.1371/journal.pone.0352451 (PMC13318055; doi:10.1371/journal.pone.0352451)
Supplement: S1 Table — (DOCX) [file pone.0352451.s001.docx]

**Table S1:** Macitentan and AI-generated derivatives with corresponding IUPAC names and 3D structures.

| Compound | IUPAC Name | 2D Structure | 3D Structure |
| --- | --- | --- | --- |
| AI Derivative 1 | (R)-N1-[1-(4-chlorophenyl)-1-oxohexan-2-yl]-N2-[1-(pyrimidin-2-yl)-1H-pyrazol-4-yl]oxalamide | 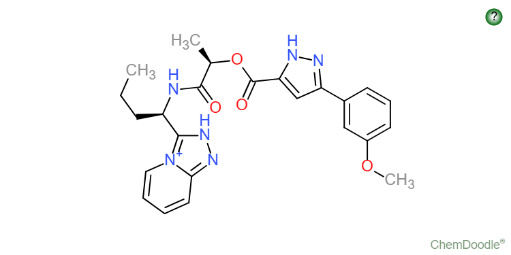 | 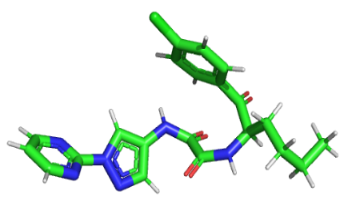 |
| AI Derivative 2 | N1-(5-bromopyridin-2-yl)-N1-butyl-N2-[4-methoxy-3-(pyridin-2-ylmethoxy)phenyl]oxalamide | 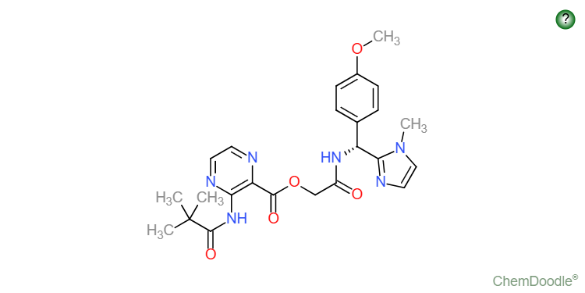 | 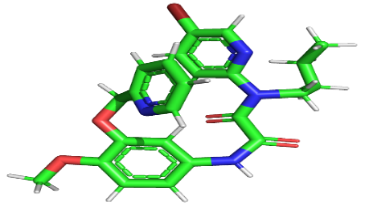 |
| AI Derivative 3 | Methyl 2-[N-butyl-2-oxo-2-({6-[(pyridin-3-yloxy)]pyridin-3-yl}amino)acetamido]-3-methylbenzoate | 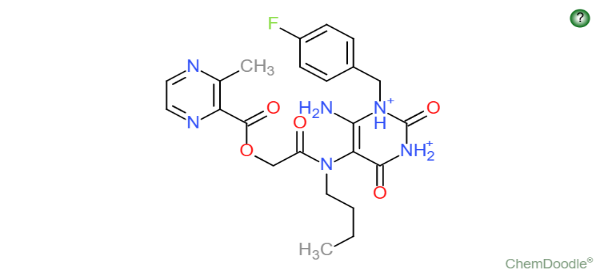 | 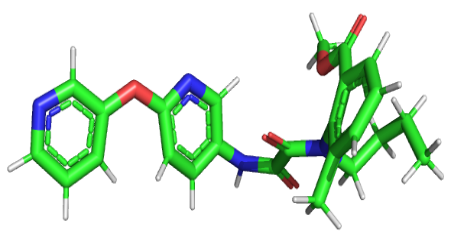 |
| AI Derivative 4 | (R)-1-Oxo-1-{[(R)-1-(pyridin-2-yl)butyl]amino}propan-2-yl 5-hydroxy-2-[3-(trifluoromethyl)phenyl]-2H-1,2,3-triazole-4-carboxylate | 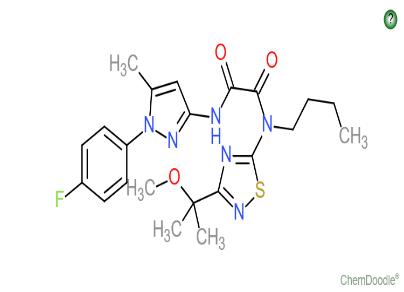 | 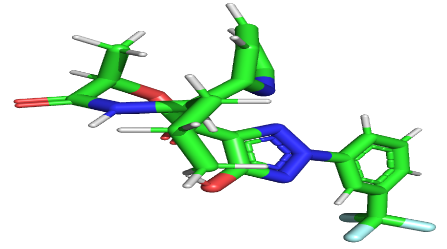 |
| AI Derivative 5 | N1-Butyl-N1-(4-methylthiazol-2-yl)-N2-[6-(pyridin-3-yloxy)pyridin-3-yl]oxalamide | 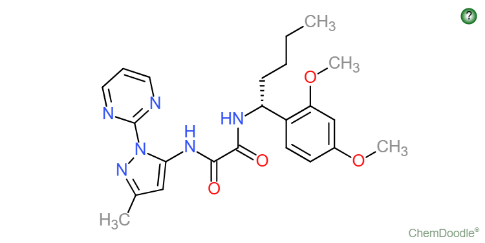 | 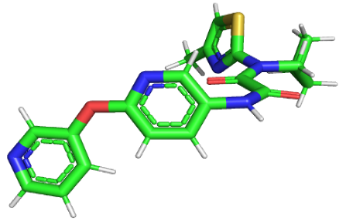 |
| AI Derivative 6 | 2-{Butyl(5-fluoropyridin-2-yl)amino}-2-oxoethyl 5-chloro-2-[(furan-2-ylmethyl)amino]pyrimidine-4-carboxylate | 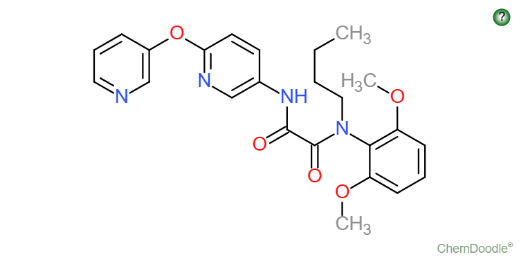 | 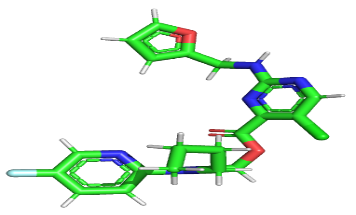 |
| AI Derivative 7 | N1-Butyl-N2-[3-fluoro-4-((6-methylpyridazin-3-yl)oxy)phenyl]-N1-(4-methylbenzyl)oxalamide | 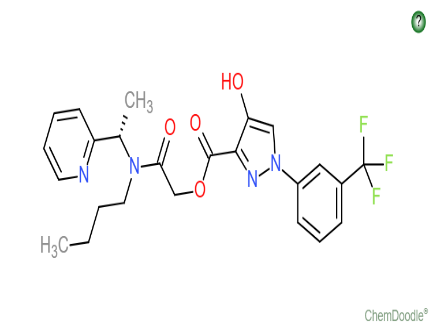 | 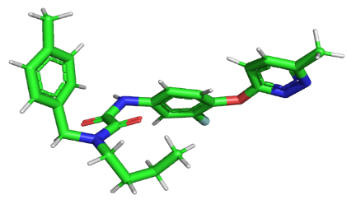 |
| AI Derivative 8 | N1-Butyl-N2-[4-methoxy-3-(5-methyl-1H-tetrazol-1-yl)phenyl]-N1-(4,5,6,7-tetrahydrobenzo[d]thiazol-2-yl)oxalamide | 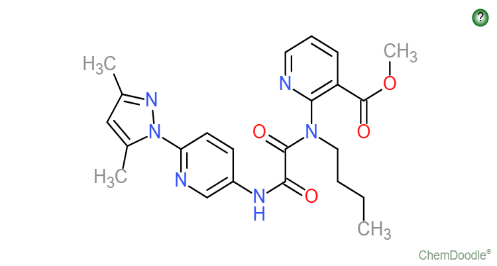 | 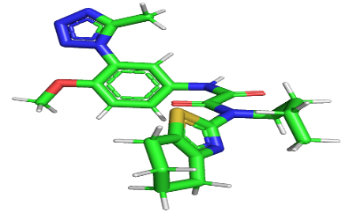 |
| AI Derivative 9 | (R)-N1-[1-(2,3-dimethoxyphenyl)pentyl]-N2-[6-methyl-2-(pyridin-4-yl)pyrimidin-4-yl]oxalamide | 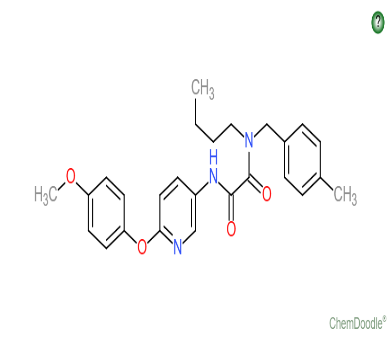 | 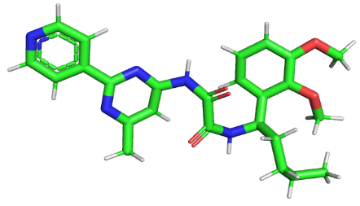 |
| AI Derivative 10 | N1-[1-(5-bromo-4-fluoropyrimidin-2-yl)-1H-pyrazol-3-yl]-N2-butyl-N2-(4-methylbenzyl)oxalamide | 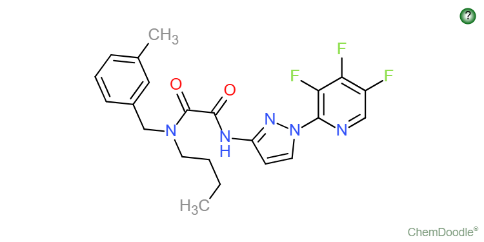 | 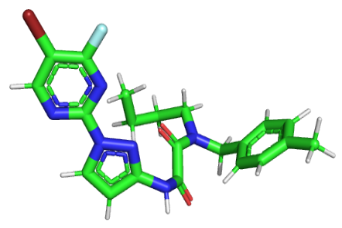 |
| AI Derivative 11 | (S)-2-{Butyl[1-(4-fluorophenyl)ethyl]amino}-2-oxoethyl 5-methyl-1H-[1,3'-bi(1,2,4-triazole)]-3-carboxylate | 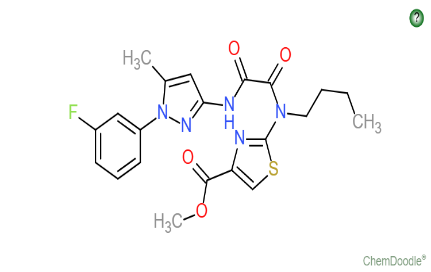 | 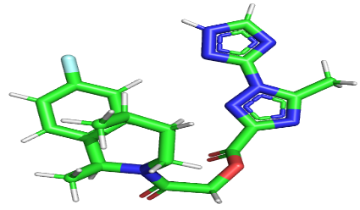 |
| AI Derivative 12 | (R)-1-{[(R)-1-(2H-tetrazol-5-yl)pentyl]amino}-1-oxopropan-2-yl 3-(3-methoxyphenyl)isoxazole-5-carboxylate | 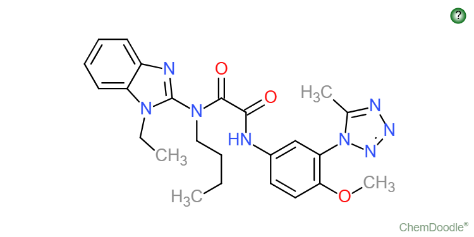 | 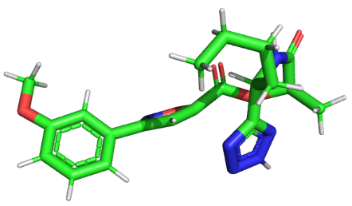 |
| AI Derivative 13 | N1-Butyl-N1-(1-ethyl-1H-benzo[d]imidazol-2-yl)-N2-[4-methoxy-3-(5-methyl-1H-tetrazol-1-yl)phenyl]oxalamide | 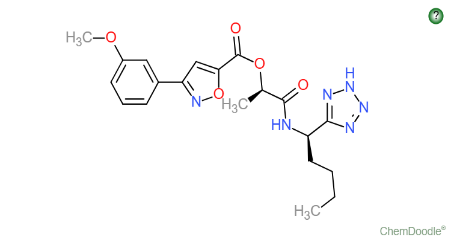 | 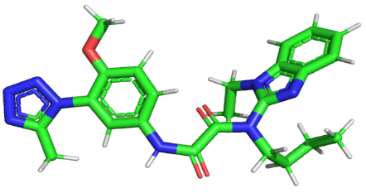 |
| AI Derivative 14 | Methyl 2-[N-butyl-2-({1-(3-fluorophenyl)-5-methyl-1H-pyrazol-3-yl}amino)-2-oxoacetamido]thiazole-4-carboxylate | 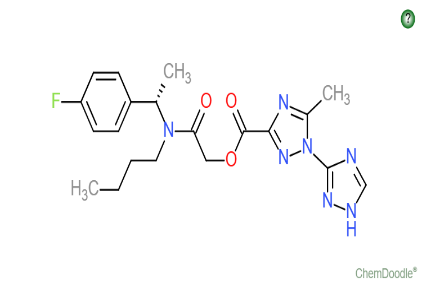 | 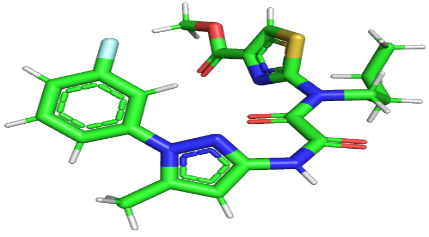 |
| AI Derivative 15 | N1-Butyl-N1-(3-methylbenzyl)-N2-[1-(3,4,5-trifluoropyridin-2-yl)-1H-pyrazol-3-yl]oxalamide | 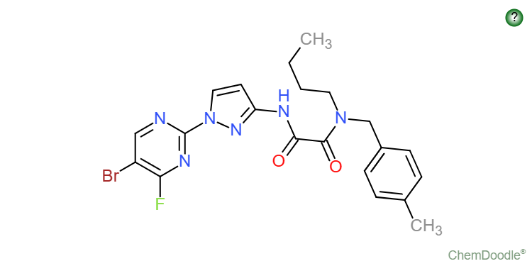 | 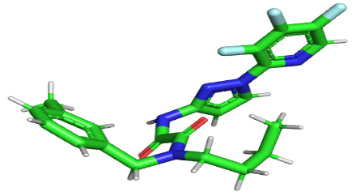 |
| AI Derivative 16 | N1-Butyl-N2-[6-(4-methoxyphenoxy)pyridin-3-yl]-N1-(4-methylbenzyl)oxalamide | 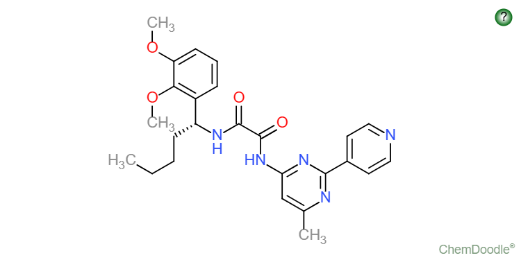 | 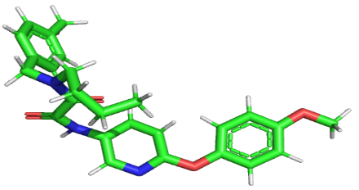 |
| AI Derivative 17 | Methyl 2-[N-butyl-2-({6-(3,5-dimethyl-1H-pyrazol-1-yl)pyridin-3-yl}amino)-2-oxoacetamido]nicotinate | 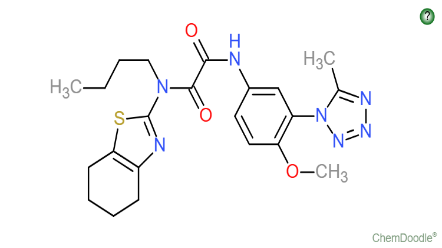 | 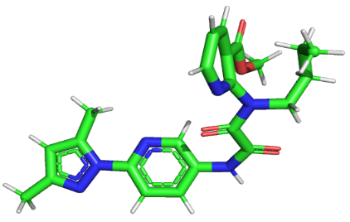 |
| AI Derivative 18 | (S)-2-{Butyl[1-(pyridin-2-yl)ethyl]amino}-2-oxoethyl 4-hydroxy-1-[3-(trifluoromethyl)phenyl]-1H-pyrazole-3-carboxylate | 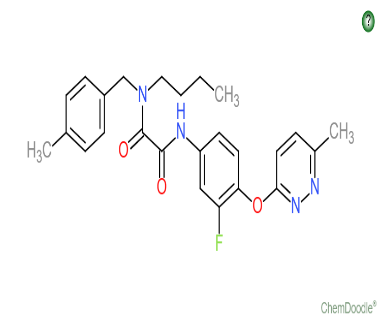 | 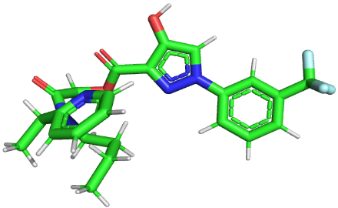 |
| AI Derivative 19 | N1-Butyl-N1-(2,6-dimethoxyphenyl)-N2-[6-(pyridin-3-yloxy)pyridin-3-yl]oxalamide | 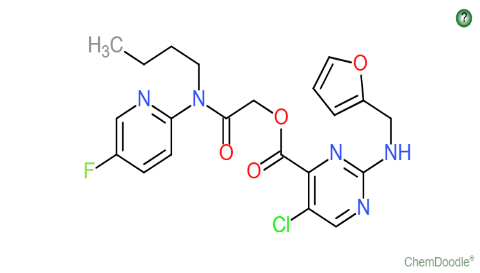 | 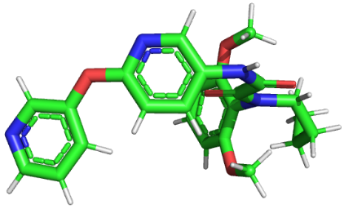 |
| AI Derivative 20 | (R)-N1-[1-(2,4-dimethoxyphenyl)pentyl]-N2-[3-methyl-1-(pyrimidin-2-yl)-1H-pyrazol-5-yl]oxalamide | 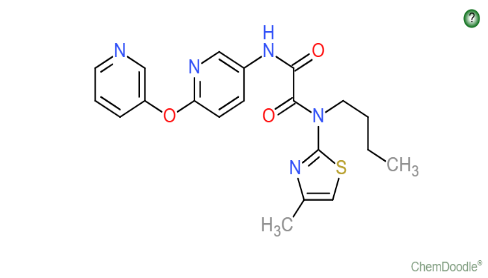 | 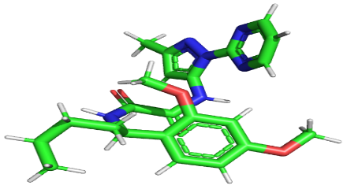 |
| AI Derivative 21 | N1-Butyl-N2-[1-(4-fluorophenyl)-5-methyl-1H-pyrazol-3-yl]-N1-[3-(2-methoxypropan-2-yl)-1,2,4-thiadiazol-5-yl]oxalamide | 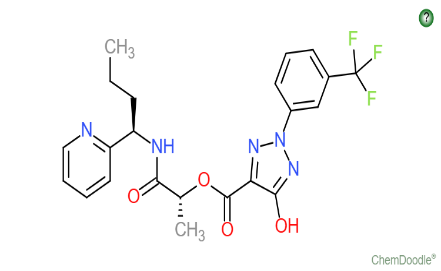 | 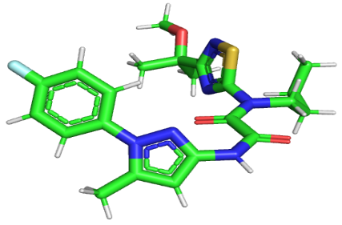 |
| AI Derivative 22 | 6-Amino-5-[N-butyl-2-((3-methylpyrazine-2-carbonyl)oxy)acetamido]-1-(4-fluorobenzyl)-2,4-dioxo-1,2,3,4-tetrahydropyrimidine-1,3-diium | 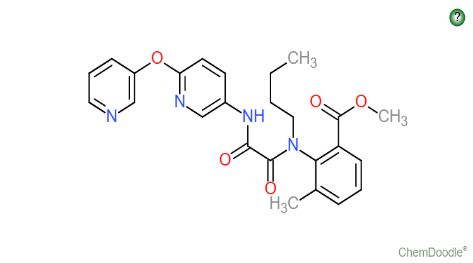 | 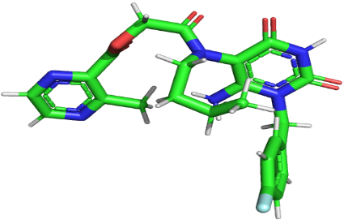 |
| AI Derivative 23 | (R)-2-{[(4-methoxyphenyl)(1-methyl-1H-imidazol-2-yl)methyl]amino}-2-oxoethyl 3-pivalamidopyrazine-2-carboxylate | 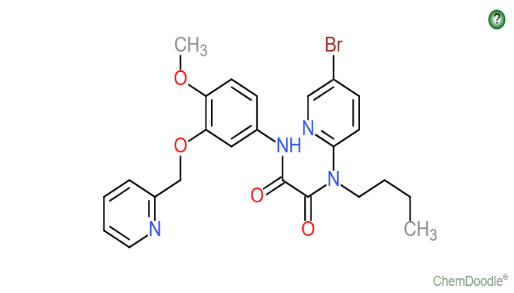 | 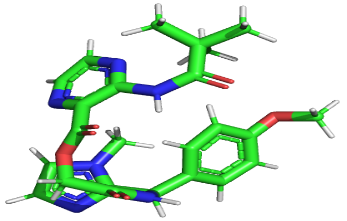 |
| AI Derivative 24 | 3-{(R)-1-[(R)-2-((3-(3-methoxyphenyl)-1H-pyrazole-5-carbonyl)oxy)propanamido]butyl}-2H-[1,2,4]triazolo[4,3-a]pyridin-4-ium | 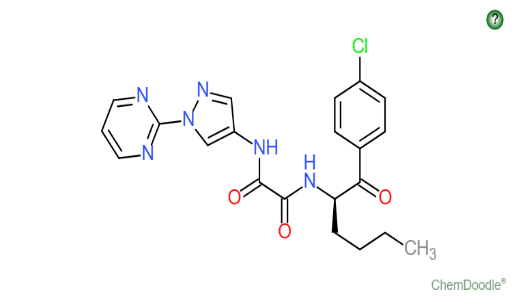 | 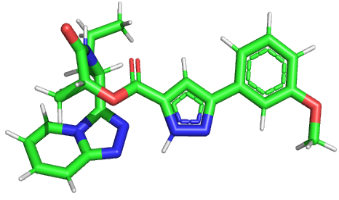 |
| Macitentan | 5-(4-Bromophenyl)-6-[2-(5-bromopyrimidin-2-yloxy)ethoxy]-N-(propylsulfamoyl)pyrimidin-4-amine | 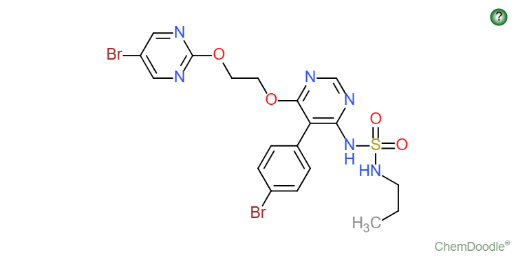 | 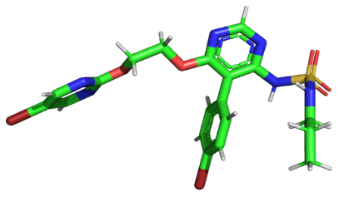 |
