# Supplementary material for: AI-driven molecular diversification and ligand-based optimization of macitentan derivatives targeting VEGFR1 and endothelin signaling pathways
Source: PLoS One. 2026 Jun 30;21(6):e0352451. doi: 10.1371/journal.pone.0352451 (PMC13318055; doi:10.1371/journal.pone.0352451)
Supplement: Attachment — Submitted filename: Response_R1.docx [file pone.0352451.s002.docx]

**Point by Point Response**

**We sincerely thank the Editor and the Reviewers for their valuable time, careful evaluation, and constructive comments on our manuscript. We greatly appreciate the insightful suggestions, which have helped us substantially improve the scientific quality, clarity, organization, and presentation of the manuscript. All comments were carefully considered, and the manuscript has been thoroughly revised accordingly. Detailed point-by-point responses to each comment are provided below, and all corresponding modifications have been incorporated into the revised version of the manuscript.**

**Reviewer 1 comments:**

1. Include a list of abbreviations found in the manuscript and arranged alphabetically.

**Response: Thank you for this valuable suggestion. A comprehensive list of abbreviations used throughout the manuscript has now been added in alphabetical order to improve readability and assist readers in navigating the technical terminology used in the study.**

2. Add high resolution image of a graphical abstract that summarizes the whole story of your work.

**Response: Thank you for this valuable suggestion. A high-resolution graphical abstract has now been added to the revised manuscript to summarize the overall study workflow, including the disease background of preeclampsia, AI-assisted molecular diversification of the Macitentan scaffold, comprehensive in-silico evaluation pipeline, and identification of lead derivatives targeting VEGFR1 and the Endothelin-1 receptor.**

3. Improve the SAR for Macitentan and derivative 24 with VEGFR1, and for Macitentan and derivative 15 with Endothelin-1-Receptor. For instance, Figure 5 illustrates the primary structural moieties, such as pyrazolo and triazol-pyridinium moieties, along with their primary bond interactions. In contrast, Figure 7 for derivative 15 mentions the primary moieties, such as trifluoropyridine and oxalamide, along with their primary bond interactions. Give considerable attention for the structural moieties in each derivative.

**Response: Thank you for this important suggestion. The SAR discussion has been substantially revised by giving specific attention to the major structural moieties of Macitentan, AI Derivative 24, and AI Derivative 15.**

4. Provide a recommendation for the recently developed derivatives, such as derivatives 24 and 15, that have structural characteristics comparable to Macitentan and have the potential to be employed as a treatment for preeclampsia.

**Response: Thank you for this valuable suggestion. A recommendation and future therapeutic perspective regarding AI Derivative 24 and AI Derivative 15 have now been added to the revised manuscript.**

5. The article is lengthy, necessitating a brief summary of several paragraphs to prevent redundancies. Revise all.

**Response: Thank you for this important observation. The manuscript has been carefully revised to improve readability and reduce redundancy throughout the text. Multiple paragraphs were refined and summarized to improve overall flow, conciseness, and presentation quality without affecting the interpretation of the computational findings.**

**Reviewer 2 comments:**

1. The authors are requested to carefully revise the chemical nomenclature throughout the manuscript. Several IUPAC names contain heterocyclic ring systems, stereochemical descriptors, locants, and prefixes that should be properly formatted. Appropriate italicization and formatting (e.g., stereochemical descriptors such as (R), N, locants, and heterocyclic nomenclature where applicable) should be checked consistently for all compounds.

**Response: Thank you for this important observation. The chemical nomenclature throughout the manuscript has been carefully revised and standardized according to IUPAC formatting conventions. Stereochemical descriptors such as (R)/(S), locants, heterocyclic ring nomenclature, and substituent formatting were thoroughly checked and corrected for consistency across all compounds presented in the manuscript and tables.**

2. Chemical structures presented in Figure 12 are not properly formatted. The authors are requested to redraw the ChemDraw structures with appropriate alignment, bond angles, atom positioning, and uniform formatting to improve clarity and readability.

**Response: Thank you for this valuable suggestion. Figure 12 has been revised accordingly. The previously included 2D chemical structures were removed from Figure 12, and all derivative structures have now been properly redrawn and incorporated into Table 1 with improved ChemDoodle formatting, consistent alignment, corrected bond angles, optimized atom positioning, and uniform presentation to enhance overall clarity and readability throughout the manuscript.**

3. The authors are requested to correct and maintain uniform line spacing throughout the manuscript according to the journal formatting guidelines.

**Response: Thank you for this observation. The entire manuscript has been carefully reformatted, and uniform line spacing of 1.0 has now been consistently applied throughout the document according to the journal formatting requirements.**

4. The authors repeatedly define the same abbreviations/full forms throughout the manuscript. Abbreviations such as ‘ET-1’ should be defined only once at their first appearance; repeated definitions are unnecessary.

**Response: Thank you for this important observation. The manuscript has been carefully revised to ensure that all abbreviations and full forms are defined only once at their first appearance in the text. Repeated definitions, including abbreviations such as ET-1 and other technical terms, have been removed throughout the manuscript to improve consistency, readability, and adherence to scientific writing standards.**
