# Supplementary material for: AI-driven molecular diversification and ligand-based optimization of macitentan derivatives targeting VEGFR1 and endothelin signaling pathways
Source: PLoS One. 2026 Jun 30;21(6):e0352451. doi: 10.1371/journal.pone.0352451 (PMC13318055; doi:10.1371/journal.pone.0352451)
Supplement: Peer review history

# Peer review: Decision Letter 0

**DOI:** 10.1371/journal.pone.0352451.r001

**Authors:** Prasad Sanjay Dhiwar

13 May 2026

PONE-D-26-07475

AI-Driven Molecular Diversification and Ligand-Based Optimization of Macitentan Derivatives Targeting VEGFR1 and Endothelin Signaling Pathways

PLOS One

Dear Dr. Ali,

Thank you for submitting your manuscript to PLOS ONE. After careful consideration, we feel that it has merit but does not fully meet PLOS ONE’s publication criteria as it currently stands. Therefore, we invite you to submit a revised version of the manuscript that addresses the points raised during the review process.

Please submit your revised manuscript by Jun 27 2026 11:59PM. If you will need more time than this to complete your revisions, please reply to this message or contact the journal office at plosone@plos.org. When you're ready to submit your revision, log on to https://www.editorialmanager.com/pone/ and select the 'Submissions Needing Revision' folder to locate your manuscript file.

Please include the following items when submitting your revised manuscript:

- A letter that responds to each point raised by the academic editor and reviewer(s). You should upload this letter as a separate file labeled 'Response to Reviewers'.
- A marked-up copy of your manuscript that highlights changes made to the original version. You should upload this as a separate file labeled 'Revised Manuscript with Track Changes'.
- An unmarked version of your revised paper without tracked changes. You should upload this as a separate file labeled 'Manuscript'.

If you would like to make changes to your financial disclosure, please include your updated statement in your cover letter. Guidelines for resubmitting your figure files are available below the reviewer comments at the end of this letter.

If applicable, we recommend that you deposit your laboratory protocols in protocols.io to enhance the reproducibility of your results. Protocols.io assigns your protocol its own identifier (DOI) so that it can be cited independently in the future. For instructions see: https://journals.plos.org/plosone/s/submission-guidelines#loc-laboratory-protocols. Additionally, PLOS ONE offers an option for publishing peer-reviewed Lab Protocol articles, which describe protocols hosted on protocols.io. Read more information on sharing protocols at https://plos.org/protocols?utm_medium=editorial-email&utm_source=authorletters&utm_campaign=protocols.

As the corresponding author, your ORCID iD is verified in the submission system and will appear in the published article. PLOS supports the use of ORCID, and we encourage all coauthors to register for an ORCID iD and use it as well. Please encourage your coauthors to verify their ORCID iD within the submission system before final acceptance, as unverified ORCID iDs will not appear in the published article. *Only*  the individual author can complete the verification step; PLOS staff *cannot*  verify ORCID iDs on behalf of authors.

We look forward to receiving your revised manuscript.

Kind regards,

Prasad Sanjay Dhiwar, PhD

Academic Editor

PLOS One

Journal Requirements:

When submitting your revision, we need you to address these additional requirements.

1.Please ensure that your manuscript meets PLOS ONE's style requirements, including those for file naming. The PLOS ONE style templates can be found at

https://journals.plos.org/plosone/s/file?id=wjVg/PLOSOne_formatting_sample_main_body.pdf and

https://journals.plos.org/plosone/s/file?id=ba62/PLOSOne_formatting_sample_title_authors_affiliations.pdf

2. Please note that PLOS One has specific guidelines on code sharing for submissions in which author-generated code underpins the findings in the manuscript. In these cases, we expect all author-generated code to be made available without restrictions upon publication of the work. Please review our guidelines at https://journals.plos.org/plosone/s/materials-and-software-sharing#loc-sharing-code and ensure that your code is shared in a way that follows best practice and facilitates reproducibility and reuse.

3. When completing the data availability statement of the submission form, you indicated that you will make your data available on acceptance. We strongly recommend all authors decide on a data sharing plan before acceptance, as the process can be lengthy and hold up publication timelines. Please note that, though access restrictions are acceptable now, your entire data will need to be made freely accessible if your manuscript is accepted for publication. This policy applies to all data except where public deposition would breach compliance with the protocol approved by your research ethics board. If you are unable to adhere to our open data policy, please kindly revise your statement to explain your reasoning and we will seek the editor's input on an exemption. Please be assured that, once you have provided your new statement, the assessment of your exemption will not hold up the peer review process.

4. If the reviewer comments include a recommendation to cite specific previously published works, please review and evaluate these publications to determine whether they are relevant and should be cited. There is no requirement to cite these works unless the editor has indicated otherwise.

Additional Editor Comments:

Reviewer 1 comments:

Include a list of abbreviations found in the manuscript and arranged alphabetically.

Add high resolution image of a graphical abstract that summarizes the whole story of your work.

Improve the SAR for Macitentan and derivative 24 with VEGFR1, and for Macitentan and derivative 15 with Endothelin-1-Receptor. For instance, Figure 5 illustrates the primary structural moieties, such as pyrazolo and triazol-pyridinium moieties, along with their primary bond interactions. In contrast, Figure 7 for derivative 15 mentions the primary moieties, such as trifluoropyridine and oxalamide, along with their primary bond interactions. Give considerable attention for the structural moieties in each derivative.

Provide a recommendation for the recently developed derivatives, such as derivatives 24 and 15, that have structural characteristics comparable to Macitentan and have the potential to be employed as a treatment for preeclampsia.

The article is lengthy, necessitating a brief summary of several paragraphs to prevent redundancies. Revise all.

Reviewer 2 comments:

1. The authors are requested to carefully revise the chemical nomenclature throughout the manuscript. Several IUPAC names contain heterocyclic ring systems, stereochemical descriptors, locants, and prefixes that should be properly formatted. Appropriate italicization and formatting (e.g., stereochemical descriptors such as (R), N, locants, and heterocyclic nomenclature where applicable) should be checked consistently for all compounds

2. Chemical structures presented in Figure 12 are not properly formatted. The authors are requested to redraw the ChemDraw structures with appropriate alignment, bond angles, atom positioning, and uniform formatting to improve clarity and readability.

3. The authors are requested to correct and maintain uniform line spacing throughout the manuscript according to the journal formatting guidelines.

4. The authors repeatedly define the same abbreviations/full forms throughout the manuscript. Abbreviations such as ‘ET-1’ should be defined only once at their first appearance; repeated definitions are unnecessary.

[Note: HTML markup is below. Please do not edit.]

Reviewers' comments:

Reviewer's Responses to Questions

**Comments to the Author**

1. Is the manuscript technically sound, and do the data support the conclusions?

The manuscript must describe a technically sound piece of scientific research with data that supports the conclusions. Experiments must have been conducted rigorously, with appropriate controls, replication, and sample sizes. The conclusions must be drawn appropriately based on the data presented.

Reviewer #1: Yes

Reviewer #2: Yes

**********

2. Has the statistical analysis been performed appropriately and rigorously?

Reviewer #1: I Don't Know

Reviewer #2: Yes

**********

3. Have the authors made all data underlying the findings in their manuscript fully available?

The PLOS Data policy requires authors to make all data underlying the findings described in their manuscript fully available without restriction, with rare exception (please refer to the Data Availability Statement in the manuscript PDF file). The data should be provided as part of the manuscript or its supporting information, or deposited to a public repository. For example, in addition to summary statistics, the data points behind means, medians and variance measures should be available. If there are restrictions on publicly sharing data—e.g. participant privacy or use of data from a third party—those must be specified.

Reviewer #1: Yes

Reviewer #2: Yes

**********

4. Is the manuscript presented in an intelligible fashion and written in standard English?

PLOS ONE does not copyedit accepted manuscripts, so the language in submitted articles must be clear, correct, and unambiguous. Any typographical or grammatical errors should be corrected at revision, so please note any specific errors here.

Reviewer #1: Yes

Reviewer #2: Yes

**********

5. Review Comments to the Author

Please use the space provided to explain your answers to the questions above. You may also include additional comments for the author, including concerns about dual publication, research ethics, or publication ethics. (Please upload your review as an attachment if it exceeds 20,000 characters)

Reviewer #1: Include a list of abbreviations found in the manuscript and arranged alphabetically.

Add high resolution image of a graphical abstract that summarizes the whole story of your work.

Improve the SAR for Macitentan and derivative 24 with VEGFR1, and for Macitentan and derivative 15 with Endothelin-1-Receptor. For instance, Figure 5 illustrates the primary structural moieties, such as pyrazolo and triazol-pyridinium moieties, along with their primary bond interactions. In contrast, Figure 7 for derivative 15 mentions the primary moieties, such as trifluoropyridine and oxalamide, along with their primary bond interactions. Give considerable attention for the structural moieties in each derivative.

Provide a recommendation for the recently developed derivatives, such as derivatives 24 and 15, that have structural characteristics comparable to Macitentan and have the potential to be employed as a treatment for preeclampsia.

The article is lengthy, necessitating a brief summary of several paragraphs to prevent redundancies. Revise all.

Reviewer #2: 1. The authors are requested to carefully revise the chemical nomenclature throughout the manuscript. Several IUPAC names contain heterocyclic ring systems, stereochemical descriptors, locants, and prefixes that should be properly formatted. Appropriate italicization and formatting (e.g., stereochemical descriptors such as (R), N, locants, and heterocyclic nomenclature where applicable) should be checked consistently for all compounds

2. Chemical structures presented in Figure 12 are not properly formatted. The authors are requested to redraw the ChemDraw structures with appropriate alignment, bond angles, atom positioning, and uniform formatting to improve clarity and readability.

3. The authors are requested to correct and maintain uniform line spacing throughout the manuscript according to the journal formatting guidelines.

4. The authors repeatedly define the same abbreviations/full forms throughout the manuscript. Abbreviations such as ‘ET-1’ should be defined only once at their first appearance; repeated definitions are unnecessary.

**********

6. PLOS authors have the option to publish the peer review history of their article (what does this mean?). If published, this will include your full peer review and any attached files.

If you choose “no”, your identity will remain anonymous but your review may still be made public.

**Do you want your identity to be public for this peer review?**  For information about this choice, including consent withdrawal, please see our Privacy Policy.

Reviewer #1: No

Reviewer #2: No

**********

[NOTE: If reviewer comments were submitted as an attachment file, they will be attached to this email and accessible via the submission site. Please log into your account, locate the manuscript record, and check for the action link "View Attachments". If this link does not appear, there are no attachment files.]

To ensure your figures meet our technical requirements, please review our figure guidelines: https://journals.plos.org/plosone/s/figures

You may also use PLOS’s free figure tool, NAAS, to help you prepare publication quality figures: https://journals.plos.org/plosone/s/figures#loc-tools-for-figure-preparation.

NAAS will assess whether your figures meet our technical requirements by comparing each figure against our figure specifications.

# Author comment: Author response to Decision Letter 1

**DOI:** 10.1371/journal.pone.0352451.r002

19 May 2026

Reviewer 1 comments:

1. Include a list of abbreviations found in the manuscript and arranged alphabetically.

Response: Thank you for this valuable suggestion. A comprehensive list of abbreviations used throughout the manuscript has now been added in alphabetical order to improve readability and assist readers in navigating the technical terminology used in the study.

2. Add high resolution image of a graphical abstract that summarizes the whole story of your work.

Response: Thank you for this valuable suggestion. A high-resolution graphical abstract has now been added to the revised manuscript to summarize the overall study workflow, including the disease background of preeclampsia, AI-assisted molecular diversification of the Macitentan scaffold, comprehensive in-silico evaluation pipeline, and identification of lead derivatives targeting VEGFR1 and the Endothelin-1 receptor.

3. Improve the SAR for Macitentan and derivative 24 with VEGFR1, and for Macitentan and derivative 15 with Endothelin-1-Receptor. For instance, Figure 5 illustrates the primary structural moieties, such as pyrazolo and triazol-pyridinium moieties, along with their primary bond interactions. In contrast, Figure 7 for derivative 15 mentions the primary moieties, such as trifluoropyridine and oxalamide, along with their primary bond interactions. Give considerable attention for the structural moieties in each derivative.

Response: Thank you for this important suggestion. The SAR discussion has been substantially revised by giving specific attention to the major structural moieties of Macitentan, AI Derivative 24, and AI Derivative 15.

4. Provide a recommendation for the recently developed derivatives, such as derivatives 24 and 15, that have structural characteristics comparable to Macitentan and have the potential to be employed as a treatment for preeclampsia.

Response: Thank you for this valuable suggestion. A recommendation and future therapeutic perspective regarding AI Derivative 24 and AI Derivative 15 have now been added to the revised manuscript.

5. The article is lengthy, necessitating a brief summary of several paragraphs to prevent redundancies. Revise all.

Response: Thank you for this important observation. The manuscript has been carefully revised to improve readability and reduce redundancy throughout the text. Multiple paragraphs were refined and summarized to improve overall flow, conciseness, and presentation quality without affecting the interpretation of the computational findings.

Reviewer 2 comments:

1. The authors are requested to carefully revise the chemical nomenclature throughout the manuscript. Several IUPAC names contain heterocyclic ring systems, stereochemical descriptors, locants, and prefixes that should be properly formatted. Appropriate italicization and formatting (e.g., stereochemical descriptors such as (R), N, locants, and heterocyclic nomenclature where applicable) should be checked consistently for all compounds.

Response: Thank you for this important observation. The chemical nomenclature throughout the manuscript has been carefully revised and standardized according to IUPAC formatting conventions. Stereochemical descriptors such as (R)/(S), locants, heterocyclic ring nomenclature, and substituent formatting were thoroughly checked and corrected for consistency across all compounds presented in the manuscript and tables.

2. Chemical structures presented in Figure 12 are not properly formatted. The authors are requested to redraw the ChemDraw structures with appropriate alignment, bond angles, atom positioning, and uniform formatting to improve clarity and readability.

Response: Thank you for this valuable suggestion. Figure 12 has been revised accordingly. The previously included 2D chemical structures were removed from Figure 12, and all derivative structures have now been properly redrawn and incorporated into Table 1 with improved ChemDoodle formatting, consistent alignment, corrected bond angles, optimized atom positioning, and uniform presentation to enhance overall clarity and readability throughout the manuscript.

3. The authors are requested to correct and maintain uniform line spacing throughout the manuscript according to the journal formatting guidelines.

Response: Thank you for this observation. The entire manuscript has been carefully reformatted, and uniform line spacing of 1.0 has now been consistently applied throughout the document according to the journal formatting requirements.

4. The authors repeatedly define the same abbreviations/full forms throughout the manuscript. Abbreviations such as ‘ET-1’ should be defined only once at their first appearance; repeated definitions are unnecessary.

Response: Thank you for this important observation. The manuscript has been carefully revised to ensure that all abbreviations and full forms are defined only once at their first appearance in the text. Repeated definitions, including abbreviations such as ET-1 and other technical terms, have been removed throughout the manuscript to improve consistency, readability, and adherence to scientific writing standards.

Attachment Submitted filename: Response_R1.docx

# Editor report: Decision Letter 1

**DOI:** 10.1371/journal.pone.0352451.r003

**Authors:** Prasad Sanjay Dhiwar

10 Jun 2026

AI-Driven Molecular Diversification and Ligand-Based Optimization of Macitentan Derivatives Targeting VEGFR1 and Endothelin Signaling Pathways

PONE-D-26-07475R1

Dear Dr. Ali,

We’re pleased to inform you that your manuscript has been judged scientifically suitable for publication and will be formally accepted for publication once it meets all outstanding technical requirements.

Within one week, you’ll receive an e-mail detailing the required amendments. When these have been addressed, you’ll receive a formal acceptance letter and your manuscript will be scheduled for publication.

An invoice will be generated when your article is formally accepted. Please note, if your institution has a publishing partnership with PLOS and your article meets the relevant criteria, all or part of your publication costs will be covered. Please make sure your user information is up-to-date by logging into Editorial Manager at Editorial Manager® and clicking the ‘Update My Information' link at the top of the page. For questions related to billing, please contact billing support.

If your institution or institutions have a press office, please notify them about your upcoming paper to help maximize its impact. If they’ll be preparing press materials, please inform our press team as soon as possible -- no later than 48 hours after receiving the formal acceptance. Your manuscript will remain under strict press embargo until 2 pm Eastern Time on the date of publication. For more information, please contact onepress@plos.org.

Kind regards,

Prasad Sanjay Dhiwar, PhD

Academic Editor

PLOS One

Additional Editor Comments (optional):

Reviewers' comments:

# Editor report: Acceptance letter

**DOI:** 10.1371/journal.pone.0352451.r004

**Authors:** Prasad Sanjay Dhiwar

PONE-D-26-07475R1

PLOS One

Dear Dr. Ali,

I'm pleased to inform you that your manuscript has been deemed suitable for publication in PLOS One. Congratulations! Your manuscript is now being handed over to our production team.

At this stage, our production department will prepare your paper for publication. This includes ensuring the following:

* All references, tables, and figures are properly cited

* All relevant supporting information is included in the manuscript submission,

* There are no issues that prevent the paper from being properly typeset

You will receive further instructions from the production team, including instructions on how to review your proof when it is ready. Please keep in mind that we are working through a large volume of accepted articles, so please give us a few days to review your paper and let you know the next and final steps.

Lastly, if your institution or institutions have a press office, please let them know about your upcoming paper now to help maximize its impact. If they'll be preparing press materials, please inform our press team within the next 48 hours. Your manuscript will remain under strict press embargo until 2 pm Eastern Time on the date of publication. For more information, please contact onepress@plos.org.

You will receive an invoice from PLOS for your publication fee after your manuscript has reached the completed accept phase. If you receive an email requesting payment before acceptance or for any other service, this may be a phishing scheme. Learn how to identify phishing emails and protect your accounts at https://explore.plos.org/phishing.

If we can help with anything else, please email us at customercare@plos.org.

Thank you for submitting your work to PLOS ONE and supporting open access.

Kind regards,

PLOS ONE Editorial Office Staff

on behalf of

Dr. Prasad Sanjay Dhiwar

Academic Editor

PLOS One
